# Kidney health outcomes in children born very prematurely compared to full-term counterparts: a systematic review and meta-analysis

**DOI:** 10.1007/s00467-025-06797-z

**Published:** 2025-05-26

**Authors:** Vaia Dokousli, Anastasia Stoimeni, Nikolaos Gkiourtzis, Despoina Samourkasidou, Vera Karatisidou, Nikolaos Charitakis, Kali Makedou, Christos Tsakalidis, George Koliakos, Despoina Tramma

**Affiliations:** 1 4th Department of Pediatrics, School of Medicine, Department of Health Sciences, “G. Papageorgiou” General Hospital, Aristotle University of Thessaloniki, 54124 & Ring Road Municipality of Pavlou Mela Area N. Evkarpia, 56403 Thessaloniki, Greece; 2 1st Department of Nephrology, School of Medicine, Department of Health Sciences, Hippokration General Hospital, Aristotle University of Thessaloniki, Thessaloniki, Greece; 3 Laboratory of Biochemistry, School of Medicine, Department of Health Sciences, AHEPA University Hospital, Aristotle University of Thessaloniki, Thessaloniki, Greece; 4 2nd Neonatal Department and Neonatal Intensive Care Unit, “G. Papageorgiou” General Hospital, School of Medicine, Department of Health Sciences, Aristotle University of Thessaloniki, Thessaloniki, Greece; 5 Laboratory of Biological Chemistry, School of Medicine, Department of Health Sciences, Aristotle University of Thessaloniki, Thessaloniki, Greece

**Keywords:** Prematurity, Biomarkers, Cystatin C, Kidney function, Children, Meta-analysis

## Abstract

**Background:**

Advances in neonatal care have improved survival rates in preterm neonates. However, concerns persist regarding the long-term kidney implications of prematurity. Nephrogenesis is disrupted, particularly in those born very preterm (≤ 32 weeks of gestation), increasing the risk of early kidney dysfunction and hypertension later in life.

**Objectives:**

This systematic review and meta-analysis aimed to evaluate kidney health outcomes in former very preterm children and adolescents compared to full-term peers.

**Data sources:**

A systematic literature search was conducted in MEDLINE/PubMed, Scopus, and Web of Science from their earliest available records to October 9, 2024.

**Study eligibility criteria:**

We included observational studies comparing kidney health parameters between children/adolescents born very preterm (gestational age – GA ≤ 32 weeks) and their full-term counterparts (gestational age > 36 weeks or birth weight > 2000 g) within the age range of 6 to 18 years.

**Participants and interventions:**

Children and adolescents aged 6–18 years born very preterm were compared to their full-term counterparts. The analyzed kidney function markers included serum Cystatin C, serum creatinine (sCr), estimated glomerular filtration rate (eGFR) based on sCr (Cr-eGFR), and blood pressure (systolic and diastolic, SBP/DBP).

**Study appraisal and synthesis methods:**

The Newcastle–Ottawa Scale was used to assess study quality. The mean difference with 95% confidence intervals was used for continuous outcomes. Statistical significance was set at *p* < 0.05. Sensitivity, subgroup and meta-regression analyses were conducted for further exploration of the outcomes. Statistical analyses were performed using R software (Version 4.3.2).

**Results:**

Thirteen studies (16 reports; 2,112 participants) were included. Very preterm children and adolescents had higher serum Cystatin C (0.05 mg/L; 95%CI: 0.02–0.08), lower Cr-eGFR (-11.87 mL/min/1.73 m^2^; 95%CI: -22.44 to -1.31), and higher SBP (1.96 mmHg; 95%CI: 0.21–3.71). Sensitivity analysis confirmed Cystatin C findings but rendered Cr-eGFR and SBP differences non-significant. Subgroup analysis showed a significant GA effect on sCr (*p* < 0.0001), though the ≥ 28 weeks subgroup included only two studies.

**Limitations:**

Considerable heterogeneity across studies persisted despite sensitivity and subgroup analyses. The lack of randomized controlled trials and longitudinal studies limits result interpretation, while non-significant meta-regression findings hinder full explanation of heterogeneity. Insufficient data prevented the assessment of additional kidney function parameters.

**Conclusions and implications of key findings:**

Cystatin C was elevated in very preterm individuals compared to full-term peers, reinforcing its role as an early marker of kidney dysfunction. While differences in Cr-eGFR and SBP lost significance after sensitivity analysis, these markers remain relevant for long-term follow-up in this vulnerable population.

**Systematic review registration number:**

PROSPERO (CRD42024554702).

**Graphical abstract:**

**Figure:**
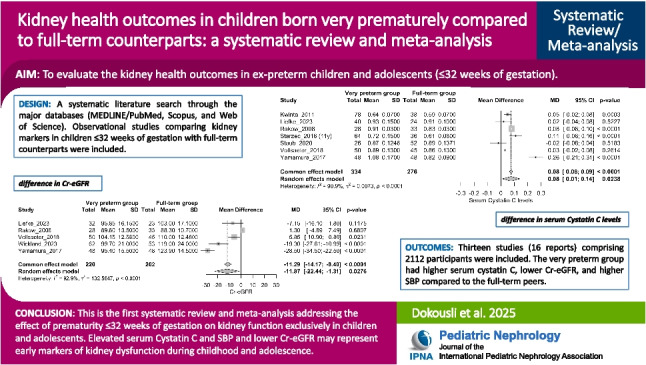
A higher resolution version of the Graphical abstract is available as Supplementary information

**Supplementary Information:**

The online version contains supplementary material available at 10.1007/s00467-025-06797-z.

## Introduction

Prematurity, defined by the World Health Organization (WHO) as birth before 37 weeks of gestation [1], affects approximately 11% of live births annually worldwide and is increasing, particularly in low- and middle-income countries. Among all preterm births, approximately 15% involve neonates born very preterm (28–32 weeks) or extremely preterm (< 28 weeks) [2]. Advances in neonatal care have improved survival rates, allowing increasingly premature infants to survive and reach adulthood [3, 4]. However, this success raises questions about the long-term health implications of preterm birth, especially for infants born at the threshold of viability [5–7]. Such considerations align with the *Developmental Origins of Health and Disease hypothesis*, which posits that early-life environmental factors, including in-utero conditions, can program long-term health outcomes, predisposing individuals to chronic diseases like hypertension, diabetes, and chronic kidney disease (CKD) later in life [8, 9].

Kidney outcomes warrant attention in preterm infants, as their progression often remains subclinical for an extended period. This focus arises because, in humans, nephrogenesis begins at 5 weeks of gestation (formation of the metanephros) and continues until 34–36 weeks, with over 60% of nephrons forming in the last trimester of pregnancy [10, 11]. Preterm birth interrupts this process, especially in infants born before 32–33 weeks [4, 10]. Autopsy studies suggest nephrogenesis may continue postnatally for up to 40 days, leading to suboptimal nephrogenesis compared to term-born counterparts [12, 13]. This decreased nephron number is worsened by nephrotoxic medications or adverse events in neonatal intensive care units (NICUs) [11]. *Brenner's hypothesis* suggests that a low nephron number increases the risk of hypertension and CKD through compensatory hypertrophy and premature nephron degeneration [14].

Studies have linked preterm birth and low birth weight (LBW) to increased risk of CKD and hypertension in adulthood [15–19]. A subset of adult CKD cases can now be attributed to prematurity. Yet, the mechanisms and timing of kidney changes during childhood and adolescence remain unclear as available studies report inconsistent results on kidney function parameters [20–35]. To date, no meta-analysis has examined the kidney health outcomes of prematurity exclusively in children and adolescents. This systematic review and meta-analysis addresses how prematurity, particularly ≤ 32 weeks of gestation, impacts kidney health in the above age range.

## Methods

### Study registration and search strategy

This systematic review and meta-analysis was performed under the Cochrane Handbook for Systematic Reviews of Interventions [36]. The reporting adhered to the guidelines set by the Preferred Reporting Items for Systematic Reviews and Meta-Analysis (PRISMA) statement [37]. A prespecified protocol has been registered in PROSPERO (CRD42024554702).

Three electronic databases—PubMed, Scopus, and Web of Science—were systematically searched for articles published on or before October 9, 2024. The search was conducted using the following terms: (children OR child OR childhood OR adolescent OR adolescents OR teenager OR teenagers OR pediatric OR pediatrics) AND [(Premature Birth) OR (Infant, Low Birth Weight) OR premature OR prematurity OR preterm OR (low birth weight)] AND [(Term Birth) OR term OR (normal BW) OR (normal birth weight) OR normal OR normative)] AND [(Glomerular Filtration Rate) OR renal OR kidney OR creatinine OR (filtration rate) OR (blood pressure)]. In PubMed, MeSH terms were additionally incorporated to enhance precision. A detailed search strategy is provided in the PROSPERO protocol. Only studies published in English were included in our study.

### Eligibility criteria

We a priori restricted inclusion to observational studies (prospective or retrospective cohort or cross-sectional) that met the following eligibility criteria: studies comparing kidney health parameters; between preterm children or adolescents with gestational age (GA) ≤ 32 weeks and their full-term peers; within the age range of 6–18 years; both groups being apparently healthy at the time of assessment; allowing for the inclusion of studies where a proportion of individuals, either preterm or full-term, were small for gestational age (SGA) or suffered intrauterine (fetal) growth restriction (IUGR/FGR); and singletons or multiples. In addition, studies were included only if they reported any of the clinical or laboratory outcomes of interest.

Based on the current literature, the preterm threshold of 32 weeks of gestation was selected as a reliable cutoff where the process of nephrogenesis is definitively disrupted [4, 10]. By agreement among the authors, studies that did not explicitly report a GA limit of 32 weeks for the very preterm group were still considered eligible if the mean GA plus 2 standard deviations met this threshold. Similarly, GA > 36 weeks or birth weight (BW) > 2000 g were deemed adequate proxies for term birth. SGA was accepted as defined by a birth weight below the 10 th percentile (z-score ≈−1.28) or below 2 standard deviations, corresponding to the 2.5 th percentile, based on the respective national growth curves [38].

We excluded studies with the following characteristics: absence of a control group; participants with congenital abnormalities, urogenital malformations, severe developmental delays or physical disabilities, known kidney impairment, or CKD at the age of assessment; non-English language publications [39], small sample sizes (n < 20 in any study group) [40], and animal studies [36, 37].

### Study procedure (collection and extraction of data)

Two reviewers (V.D. and D.S.) independently conducted the literature search and data extraction. All records retrieved from the databases were imported into a reference management tool (rayan.qcri.org) and duplicates were removed [41]. The initial screening was performed by evaluating titles and abstracts, while the remaining articles underwent full-text assessment based on the eligibility criteria. Any discrepancies were resolved through discussion with a third author (N.G.) until consensus was reached. The reference lists of all included studies, previous systematic reviews, and meta-analyses, were manually screened to identify additional studies. Furthermore, ClinicalTrials.gov, PROSPERO, OSF, and"grey literature"were searched to identify unpublished, ongoing, or published studies to avoid duplication.

Data extracted from the included studies encompassed baseline characteristics [first author, publication year, study name (if applicable), country, number of participants, study groups, female sex, GA, BW, age at assessment, SGA, multiple pregnancies] and outcomes of interest. Three authors (V.D., D.S., and N.G.) independently performed this process using a pre-specified form. For missing data, corresponding authors were contacted.

### Quality assessment of the included studies

The quality of the included studies was evaluated using the modified Newcastle–Ottawa Scale which assesses three domains: population selection, group comparability, and outcome measurement [42]. Studies were categorized as low (0–3 points), medium (4–6 points), or high quality (7–9 points). Two independent working reviewers (N.C. and V.K.) assessed the risk of bias, with disagreements resolved through consensus. Publication bias was assessed using a funnel plot, where point estimates of mean differences were plotted against their standard errors, reflecting the study weights.

### Outcome measurements

The main outcomes included serum Cystatin C, serum Creatinine (sCr), creatinine-based estimated glomerular filtration rate (Cr-eGFR), systolic blood pressure (SBP), and diastolic blood pressure (DBP).

### Statistical analysis and data synthesis

A meta-analysis was performed using R software (Version 4.3.2) with “meta” package [43]. Mean differences with 95% confidence intervals (CIs) were used for continuous outcomes, and forest plots illustrated the weighted results. Small study effects, including publication bias, were assessed via Egger’s test and comparison-adjusted funnel plots [44]. Heterogeneity was evaluated using the I^2^ test [36] and sensitivity analysis (leave-one-out) examined the impact of individual studies. Baujat plots were additionally used to identify studies contributing most to heterogeneity. Subgroup analyses were performed based on mean age of participants at the time of assessment (< 10 years vs. ≥ 10 years) and mean GA of the preterm group (extremely preterm group, < 28 weeks vs. ≥ 28 weeks), while meta-regression explored their influence when feasible. Statistical significance was set at *p* < 0.05.

## Results

### Search strategy results

**Fig. 1:**
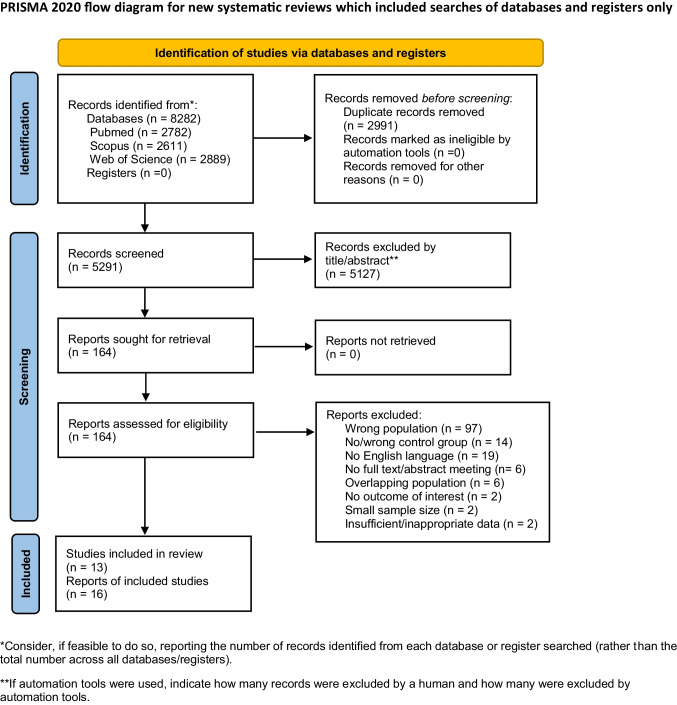
The PRISMA 2020 flow chart

The initial search identified 8,282 articles. After duplicate detection, 2991 records were removed (Fig. 1). After title and abstract screening, we assessed 164 full-text records for eligibility (Appendix Table 1). Finally, 16 reports from 13 studies were included in the systematic review [20–35].

### Study characteristics

All studies included in the systematic review and meta-analysis were observational (cross-sectional, prospective, or retrospective cohort studies) [20–35]. Table 1 presents the characteristics of the included studies. Overall, 2,112 participants were included with a median age of 10.93 years. The reports by *Centra *et al*.* [28] and *Roberts *et al*.* [29] derive from the VICS study; we used blood pressure data at 8 years of age from *Roberts *et al. (larger sample at this age) and at 18 years of age from *Centra *et al*.* (as *Roberts *et al*.* did not provide data for this age group). Similarly, the studies by *Kwinta *et al*.* [31], *Starzek *et al*.* [32], and *Gilarska *et al*.* [33] examined the same population, first at the age of 7 years old (*Kwinta *et al*.*) and later at the age 11 years old (*Starzek *et al*.* and *Gilarska *et al.), with the latter two studies focusing on distinct outcomes of interest.

**Table 1:** Baseline characteristics of included studies

Study	Country	No of participants N	Study groups of interest	n	Female n, (%)	GA Weeks	BW G	Age at assessment Years	SGA or FGR n (%)	Multiple pregnancy n (%)
Liefke 2023	Sweden	64	PT (FGR) PT (AGA) FT (AGA)	21 19 24	11 (52.4) 9 (47.4) 13 (54.2)	26.9 {24.6–29.1} 27.6 {24.4–29.7} 40.0 {38.4–40.7}	660 {395–976} 1110 {660–1790} 3445 {3000–4160}	15.00 {13.00–16.00} 15.00 {13.00–16.00} 15.00 {13.00–16.00}	21 (100.0) 0 0	NA
Wickland 2023	USA	186	PT (VLBW) FT	62 124	29 (46.8) 58 (46.8)	28.0 ± 1.8 {24.0–32.0} NA	1073 ± 251 {480–1507} 3343 ± 524	11.00 ± 1.00 11.00 ± 1.00	9 (14.5) NA	NA
Staub 2020 (BSPC Study)	Switzerland	133	PT FT	51 82	25 (49.0) 35 (42.7)	31.0 [30.7–31.9] 39.9 [39.6–40.6]	1360 [1094–1626] 3250 [2973–3528]	12.30 [11.37–13.24] 12.10 [11.50–12.70]	5 (9.8) 8 (9.8)	7 (13.7) NA
Rakow 2019	Sweden	60	PT (NC +) PT (NC-) FT	20 21 19	11 (55.0) 8 (38.1) 9 (47.4)	25.5 ± 1.2 25.9 ± 1.3 39.7 ± 1.6	755 ± 124 841 ± 202 3586 ± 477	7.80 ± 1.00 7.40 ± 1.10 8.10 ± 1.20	3 (15.0) 5 (23.8) 0	NA
Watterberg 2019 (SUPPORT Study)	USA	259	PT FT	219 40	103 (47.0) 20 (50.0)	26.4 ± 1.03 39.3 ± 0.95	877 ± 190 3463 ± 464	6.90 ± 0.43 6.70 ± 0.20	11 (5.0) 3 (7.5)	NA 0
Vollsaeter 2018	Norway	111	PT (SGA) PT (AGA) FT (AGA)	20 37 54	7 (35.0) 21 (56.8) 25 (46.3)	28.0 |27.2–28.7| 26.1 |25.7–26.5| > 37.0*	724 |657–791| 918 |867–968| 3701 |3582–3819|	11.30 [11.00–11.80] 11.40 [11.10–11.80] 11.70 [11.20–12.00]	20 (100.0) 0 0	NA
Bonamy 2017 (EXPRESS Study)	Sweden	343	PT (EPT) FT	171 172	76 (44.4) 73 (42.4)	25.4 ± 1.0 {22.9–26.9} 39.8 ± 1.2 {37.1–41.9}	786 ± 169 {348–1161} 3595 ± 465 {2430–4985}	6.60 ± 0.19 6.70 ± 0.18	22 (12.9) 3 (1.7)	32 (18.7) 0
Yamamura-Miyazaki 2016	Japan	96	PT (ELBW) FT	48 48	22 (45.8) 15 (31.3)	26.9 [25.6–28.6] 39.1 [38.9–40.0]	792 [630–872] 3040 [2829–3498]	8.30 [7.70–8.60] 8.10 [6.60–10.80]	23 (47.9) NA	18 (37.5) NA
Centra 2015 (VICS Study)	Australia	191	EPT/ELBW FT (AGA)	120 71	58 (48.3) 41 (57.7)	26.6 ± 2.0 39.1 ± 1.4	896 ± 161 3408 ± 432	8.00 & 18.00*	NA 0	NA
Roberts 2014 (VICS Study)	Australia	256	EPT FT (AGA)	136 120	65 (47.8) 69 (57.5)	25.8 ± 1.1 39.3 ± 1.4	890 ± 172 3428 ± 453	18.00*	NA 0	NA
McEniery 2011 (EPICure Study)	UK	372	EPT FT	219 153	118 (53.9) 89 (58.2)	24.9 ± 0.7 ≥ 37.0*	740 ± 120 NA	10.92 ± 0.38 10.94 ± 0.55	NA	NA
Kwinta 2011	Poland	116	PT (ELBW) FT	78 38	51 (65.4) 19 (50.0)	27.0 [26.0–29.0] 40.0 [39.0–41.0]	890 [760–950] 3545 [3409–3820]	6.70 [6.40–6.90] 6.90 [6.30–7.30]	22 (28.2) 2 (5.3)	10 (12.8) 0
Starzec 2016	Poland	100	PT (ELBW) FT	64 36	43 (67.2) 17 (47.2)	27.0 [25.0–28.0] 40.0 [39.0–41.0]	875 [750–960] 3570 [3395–3880]	11.00 [10.80–11.30] 10.70 [10.20–11.10]	19 (29.7) 2 (5.6)	9 (14.1) 0
Gilarska 2016	Poland	105	PT (ELBW) FT	67 38	44 (65.7) 19 (50.0)	27.0 ± 2.27 39.8 ± 1.42	850 ± 128 3571 ± 538	10.99 ± 0.34 10.61 ± 0.85	NA	NA
Rakow 2008	Sweden	76	PT FT (AGA)	39 37	22 (56.4) 24 (64.9)	26.6 ± 2.0 39.6 ± 1.0	954 ± 203 3485 ± 502	9.60 ± 0.30 9.80 ± 0.20	6 (15.4) 0	0
Bonamy 2007	Sweden	60	PT FT (AGA)	39 21	20 (51.3) 10 (47.6)	28.9 ± 1.6 {25.0–30.0} 40.3 ± 1.0 {38.0–42.0}	1106 ± 305 {472–1883} 3704 ± 404 {2690–4740}	9.10 ± 1.70 9.70 ± 1.50	20 (51.3) 0	0

Abbreviations: *AGA* appropriate for gestational age, *BW* birth weight, *ELBW* extremely low birth weight, *EPT* extremely preterm, *FGR* fetal growth restriction, *FT* full-term, *GA* gestational age, *PT* preterm, *VLBW* very low birth weight, *NA* not applicable, *NC* + nephrocalcinosis positive, *NC-* nephrocalcinosis negative, *SGA* small for gestational age

Data are presented as the number of participants, the number of participants with the percentage in parentheses, the median with interquartile range (IQR) in square brackets, the median with range in curly braces, the mean with 95% confidence intervals (CI) in vertical bars, the mean ± standard deviation (SD), and the range in curly braces, as appropriate

^*^Further details are unavailable

### Quality assessment of the included studies

The quality assessment of the included studies, according to the Newcastle–Ottawa Scale, is presented in Table 2. Ten studies were categorized as high quality [20, 22, 23, 25, 26, 28–31, 35] and six studies as medium quality [21, 24, 27, 32–34].

**Table 2:** Quality assessment of included studies with Newcastle–Ottawa Scale

First author & Year	Is the case definition adequate?	Representativeness of the cases	Selection of controls	Definition of controls	Comparability of cases and controls based on the design or analysis	Ascertainment of exposure	Same method of ascertainment for cases and controls	Non-response rate	Total score
Centra 2015	*	*	*	*	**	*	*		8/9
Edstedt Bonamy 2007	*	*				*	*	*	5/9
Edstedt Bonamy 2017	*	*	*	*	**	*	*		8/9
Gilarska 2016	*	*	*		*	*	*		6/9
Kwinta 2011	*	*	*		**	*	*	*	8/9
Liefke 2023	*	*	*	*	*	*	*		7/9
McEniery 2011	*	*	*		**	*	*		7/9
Rakow 2008	*		*	*	**	*	*	*	8/9
Rakow 2019	*	*		*		*	*		5/9
Roberts 2014	*	*	*		**	*	*		7/9
Starzec 2016	*	*	*		*	*	*		6/9
Staub 2020	*	*	*		**	*	*		7/9
Vollsaeter 2018	*	*	*		*	*	*	*	7/9
Watterberg 2019	*	*	*	*		*	*	*	7/9
Wickland 2023	*	*			*	*	*	*	6/9
Yamamura-Miyazaki 2017	*	*			*	*	*	*	6/9

### Analysis of outcomes

#### Serum Cystatin C

**Fig. 2:**
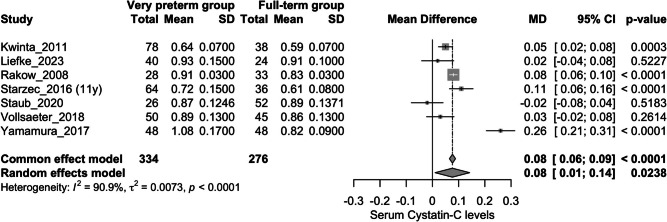
Forest plot assessing the difference in serum Cystatin C levels between very preterm group vs. full-term group

**Fig. 3:**
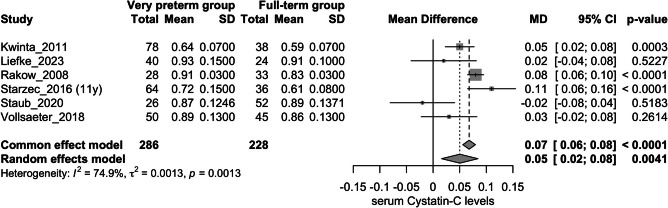
Forest plot assessing the difference in serum Cystatin C levels between very preterm group vs. full-term group after sensitivity analysis

Overall, seven studies assessed this outcome (Fig. 2) [20, 22, 23, 25, 27, 31, 32]. The very preterm group exhibited higher levels of serum Cystatin C compared to the full-term group (mean difference: 0.08 mg/L; 95%CI: 0.01 to 0.14 mg/L); however, there was substantial heterogeneity (I^2^ = 90.9%). Following a sensitivity analysis, the study by *Yamamura *et al*.* was omitted (Appendix Fig. 1) [27]. The significant contribution of this study to the overall heterogeneity was confirmed using a *Baujat plot* (Appendix Fig. 2). After this adjustment, the very preterm group still showed higher serum Cystatin C levels compared to the full-term group (mean difference: 0.05 mg/L; 95%CI: 0.02 to 0.08 mg/L), with reduced heterogeneity (I^2^ = 74.9%) (Fig. 3).

### Serum creatinine

A total of eight studies assessed this outcome [20–25, 27, 32], showing no significant difference in sCr levels between the very preterm and full-term groups (mean difference: 1.47 μmol/L; 95%CI: −2.45 to 5.39 μmol/L; I^2^ = 93.7%) (Appendix Fig. 3). Sensitivity analysis indicated that the study by *Rakow *et al*.* [23] had a high impact on heterogeneity. Its exclusion yielded similar results (mean difference: 2.47 μmol/L; 95%CI: −1.56 to 6.50 μmol/L; I^2^ = 86.0%) (Appendix Figs. 4 and Appendix Fig. 5). Publication bias assessment was not statistically significant.

A subgroup analysis considering the mean GA (< 28 weeks vs. ≥ 28 weeks) as a potential moderator was conducted. The test for subgroup differences was statistically significant (*p* = 0.01). Of note, children born at ≥ 28 weeks had higher sCr levels compared to full-term peers (mean difference: 6.90 μmol/L; 95% CI: 3.51 to 10.30 μmol/L), with zero heterogeneity (I^2^ = 0%). However, this subgroup included only two studies (Appendix Fig. 6).

### Creatinine-estimated glomerular filtration rate

**Fig. 4:**
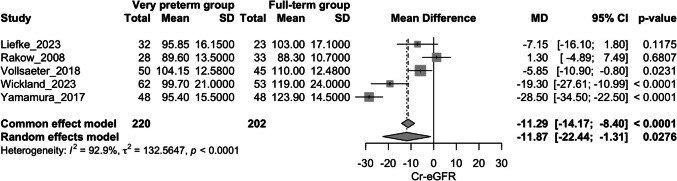
Forest plot assessing the difference in Cr-eGFR between very preterm group vs. full-term group; Cr-eGFR: Creatinine-estimated glomerular filtration rate

Five studies evaluated this outcome [20, 21, 23, 25, 27]. The very preterm group exhibited lower Cr-eGFR values compared to the full-term group (mean difference: −11.87 ml/min/1.73 m^2^; 95%CI: −22.44 to −1.31 ml/min/1.73 m^2^; I^2^ = 92.9%) (Fig. 4). After a sensitivity analysis, the study by *Yamamura *et al*.* was omitted and the difference between groups was no longer significant (mean difference: −7.42 ml/min/1.73 m^2^; 95%CI: −15.66 to 0.81 ml/min/1.73 m^2^; I^2^ = 80.3%) (Appendix Fig. 7). Subgroup analysis based on mean age (< 10 years vs. ≥ 10 years) showed no significant difference between subgroups (*p* = 0.84) (Appendix Fig. 8).

### Systolic blood pressure

**Fig. 5:**
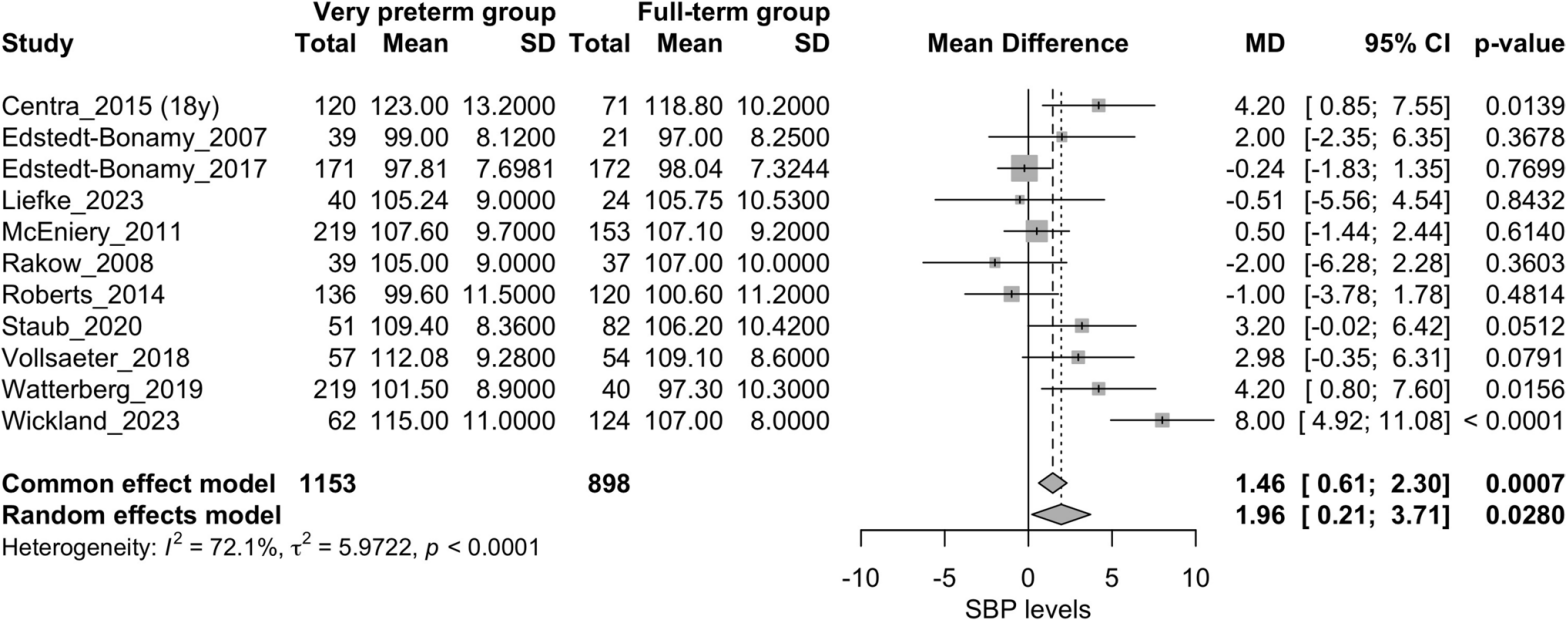
Forest plot assessing the difference in systolic blood pressure levels between very preterm group vs. full-term group; SBP: systolic blood pressure

A total of 11 studies assessed this outcome [20–23, 25, 26, 28–30, 34, 35]. The very preterm group showed higher SBP than full-term group (mean difference: 1.96 mmHg; 95%CI: 0.21 to 3.71 mmHg; I^2^ = 72.1%) (Fig. 5). Sensitivity analysis identified the study by *Wickland *et al. as a major contributor to heterogeneity. This was also validated by a *Baujat plot* (Appendix Fig. 9 and 10). After its removal, the difference was no longer significant (mean difference: 1.26 mmHg; 95%CI: −0.08 to 2.60 mmHg); I^2^ = 47.1%] (Appendix Fig. 11). Publication bias assessment was not statistically significant (Appendix Fig. 12).

Furthermore, the meta-regression analysis using a mixed-effects model revealed that the mean age of participants was not a significant predictor of effect size (*p* = 0.92) and explained none of the heterogeneity (R^2^ = 0.00%). The model’s intercept (2.2782, *p* = 0.46) was also not significant (Appendix Fig. 13). Another model incorporating mean GA as a moderator explained 18.78% of the variance. However, mean GA was not a significant moderator (*p* = 0.14), despite the positive slope (Appendix Fig. 14).

### Diastolic blood pressure

Ten studies evaluated this outcome [20, 22, 23, 25, 26, 28–30, 34, 35], showing no significant difference in DBP between the very preterm and full-term groups (mean difference: 0.62 mmHg; 95%CI: −0.50 to 1.74 mmHg; I^2^ = 52.8%) (Appendix Fig. 15). Sensitivity analysis led to the exclusion of the study by *Centra *et al., but the results remain non-significant (mean difference: 0.25 mmHg; 95%CI: −0.49 to 0.99 mmHg); I^2^ = 0%] (Appendix Fig. 16). Publication bias assessment was not statistically significant.

## Discussion

Prematurity leads to immediate, short-term, and long-term implications forcing the human body into microstructural abnormalities [10]. The kidney consequences of prematurity include a higher risk of CKD and hypertension later in life [45]. While several studies have investigated the long-term effects of prematurity, its exact impact on kidney function during childhood and adolescence remains unclear [46, 47]. This study presents the first systematic review and meta-analysis assessing the effect of very preterm birth on kidney function in this age group.

Although emerging evidence suggests that kidney development can persist postnatally after preterm birth for up to 40–62 days, prematurity is known to impair kidney function, volume, size and kidney-to-bodyweight ratio [11, 45, 47, 48]. Children born very premature should be screened at six months and not later than one year of age with urinary albumin-to-creatinine ratio (uACR), sCr-to-Cystatin C ratio, blood pressure and kidney ultrasound, to detect potential functional or structural abnormalities [49]. Cystatin C is a low-molecular-weight protein produced at a constant rate by nucleated cells, freely filtered by the glomeruli, and almost completely reabsorbed by proximal tubular cells without secretion. Unlike sCr, it is less influenced by muscle mass, age, diet, or gender [50]. It has been shown to detect early subclinical GFR changes before sCr abnormalities appear [51].

Given the variability in nephrogenesis completion (32–37 weeks GA) [11, 12, 52], we set 32 weeks as the cutoff to ensure nephrogenesis impairment in our study population. We focused on childhood and adolescence, as the impact of prematurity on kidney function may be masked by compensatory mechanisms, making it challenging to detect [46].

Our findings suggest that serum Cystatin C, Cr-eGFR, and SBP may help identify early kidney involvement in former very premature individuals. Specifically, serum Cystatin C was higher in our study group compared to controls even after a sensitivity analysis (mean difference: 0.05 mg/L; 95%CI: 0.02 to 0.08 mg/L; I^2^ = 74.9%). Additionally, the very preterm group showed lower Cr-eGFR and higher SBP levels compared to the full-term group. Following sensitivity analysis, differences in Cr-eGFR and SBP were no longer significant, while considerable heterogeneity persisted.

To further explore heterogeneity, we performed subgroup and meta-regression analyses. Subgroup analysis for sCr revealed a significant difference between GA subgroups (*p* < 0.001), indicating GA as a crucial determinant of kidney function outcomes. However, this subgroup (with I^2^ = 0%) included only two studies, limiting the generalizability of this finding. In the Cr-eGFR subgroup analysis, no significant difference was found between age groups (< 10 vs. ≥ 10 years).

Meta-regression analyses examined whether GA and age could explain heterogeneity in SBP. Both variables were not significant predictors of effect size (*p* = 0.14 and *p* = 0.92, respectively), with GA accounting for 18.78% of heterogeneity and age explaining none of it (R^2^ = 0.00%).

These findings suggest that, while GA and age may influence kidney function variability in preterm populations, additional confounders likely play a role, requiring further investigation in larger, homogeneous samples.

*Ηeo and Lee* conducted a meta-analysis on individuals born preterm (GA < 37 weeks), assessing kidney function from childhood to adulthood [47]. They reported lower GFR, reduced kidney size, and higher uACR, SBP, and DBP in preterm individuals compared to full-term controls. However, they did not find a significant difference in serum Cystatin C levels. This discrepancy may partly be attributed to our inclusion of more studies (seven vs. three), despite similar prematurity levels across analyses, regarding this outcome.

The same approach was applied in the systematic review of *Sangla and Kandasamy* [46]. The study concluded that prematurity may be linked to kidney dysfunction, CKD, and hypertension in pediatric patients and young adults. Nevertheless, it lacks the meta-analytic component limiting the thoroughness of their findings. In addition, the inclusion of preterm individuals with GA < 37 weeks, may have diluted the specific impact of very and extremely preterm birth on kidney outcomes.

Finally, *Goetschalckx *et al*.* conducted a pooled analysis showing reduced GFR across the full pediatric age range of former extremely low birth weight (ELBW) individuals. However, substantial variability in GFR measurement patterns and the absence of evaluation of other kidney function markers limit the ability to draw broader conclusions [53].

Our meta-analysis has several limitations. The lack of randomized controlled trials raises the total disparity since gestational age cannot be randomized. Additionally, some studies were excluded due to the unavailable raw data. While some included studies assessed the same population at different time points, comprehensive longitudinal studies are needed for a clearer understanding of CKD and hypertension risk in preterm-born individuals. Heterogeneity in most outcomes remained high despite sensitivity and subgroup analyses. We could not assess eGFR based on Cystatin C, kidney size, or kidney volume due to insufficient data.

Despite these limitations, our study includes a large sample (*N* = 2,112) and extensive meta-analyses. We conducted a thorough literature search to ensure comprehensive data inclusion, and most included studies were of high quality.

Our findings suggest that Cystatin C, Cr-eGFR, and SBP may serve as early markers of kidney dysfunction in very preterm children and adolescents, with Cystatin C emerging as the most reliable biomarker among the three.

## Conclusion

This study represents the first systematic review and meta-analysis exclusively examining the effect of very preterm birth on kidney function in children and adolescents. Our findings confirm the significant elevation of Cystatin C in this population, emphasizing its already-known role as an early marker of kidney dysfunction. Additionally, Cr-eGFR and SBP may warrant closer monitoring in this population. Despite limitations, the inclusion of more than 2,000 participants strengthens the robustness of our findings and underscores the need for ongoing follow-up of kidney function in former very preterm children and adolescents.

## Supplementary Information

Below is the link to the electronic supplementary material.

Graphical abstract (PPTX 370 KB)

Supplementary file2 Appendix Figure 1. Sensitivity analysis of the outcome of serum Cystatin C levels (PNG 296 KB)

Supplementary file3 Appendix Figure 2. Baujat plot for assessing the contribution of each study to overall heterogeneity in the outcome of serum Cystatin C levels (PNG 82 KB)

Supplementary file4 Appendix Figure 3. Forest plot assessing the difference of serum creatinine levels between very preterm group vs. full-term group (PNG 344 KB)

Supplementary file5 Appendix Figure 4. Sensitivity analysis of the outcome of serum creatinine levels (PNG 189 KB)

Supplementary file6 Appendix Figure 5. Forest plot assessing the difference of serum creatinine levels between very preterm group vs. full-term group after sensitivity analysis (PNG 327 KB)

Supplementary file7 Appendix Figure 6. Subgroup analysis of the outcome of serum creatinine levels by gestational age groups; GA: gestational age (PNG 510 KB)

Supplementary file8 Appendix Figure 7. Forest plot assessing the difference of Cr-eGFR between very preterm group vs. full-term group after sensitivity analysis; Cr-eGFR: Creatinine-estimated glomerular filtration rate (PNG 283 KB)

Supplementary file9 Appendix Figure 8. Subgroup analysis of the outcome of Cr-eGFR by age at the time of assessment groups; Cr-eGFR: Creatinine-estimated glomerular filtration rate (PNG 470 KB)

Supplementary file10 Appendix Figure 9. Sensitivity analysis of the outcome of systolic blood pressure; SBP: systolic blood pressure (PNG 243 KB)

Supplementary file11 Appendix Figure 10. Baujat plot for assessing the contribution of each study to overall heterogeneity in the outcome of systolic blood pressure; SBP: systolic blood pressure (PNG 91 KB)

Supplementary file12 Appendix Figure 11. Forest plot assessing the difference of systolic blood pressure levels between very preterm group vs. full-term group after sensitivity analysis; SBP: systolic blood pressure (PNG 393 KB)

Supplementary file13 Appendix Figure 12. Funnel plot for assessing publication bias (PNG 70 KB)

Supplementary file14 Appendix Figure 13. Meta-regression analysis of systolic blood pressure effect size by mean age at assessment; SBP: systolic blood pressure (PNG 67 KB)

Supplementary file15 Appendix Figure 14. Meta-regression analysis of systolic blood pressure by mean gestational age; SBP: systolic blood pressure; GA: gestational age (PNG 74 KB)

Supplementary file16 Appendix Figure 15. Forest plot assessing the difference of diastolic blood pressure levels between very preterm group vs. full-term group; DBP: diastolic blood pressure (PNG 393 KB)

Supplementary file17 Appendix Figure 16. Forest plot assessing the difference of diastolic blood pressure levels between very preterm group vs. full-term group after sensitivity analysis; DBP: diastolic blood pressure (PNG 369 KB)

Supplementary file18 (DOCX 33 KB)

Supplementary file19 (DOCX 34 KB)

## Data Availability

The datasets generated during and/or analysed during the current study are available from the corresponding author on reasonable request.
