# Supplementary material for: Kidney health outcomes in children born very prematurely compared to full-term counterparts: a systematic review and meta-analysis
Source: Pediatr Nephrol. 2025 May 26;41(1):61–72. doi: 10.1007/s00467-025-06797-z (PMC12686000; doi:10.1007/s00467-025-06797-z)
Supplement: Supplementary file 1 — Graphical abstract (PPTX 370 KB) [file 467_2025_6797_MOESM1_ESM.pptx]

## Slide 1
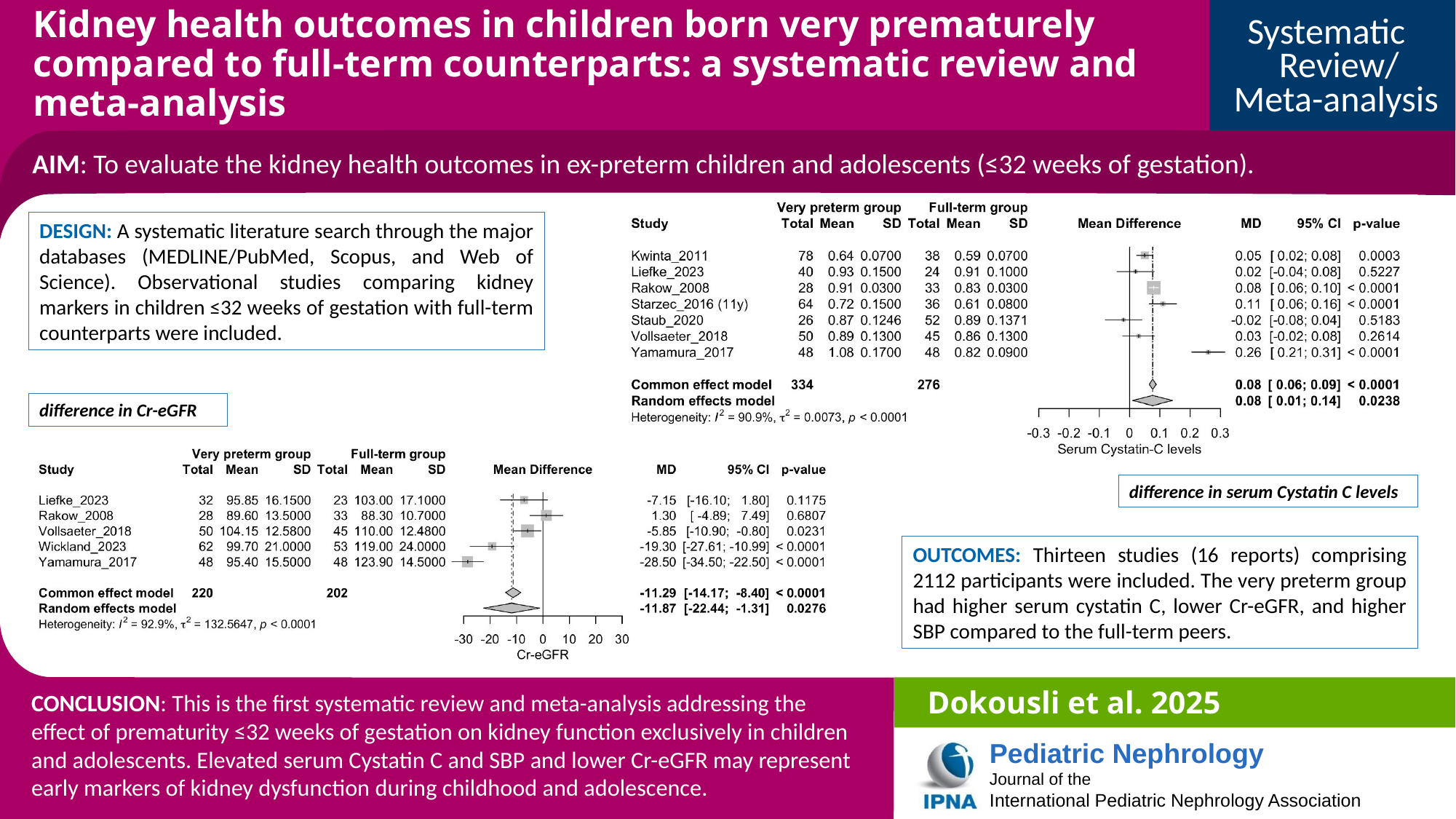

Kidney health outcomes in children born very prematurely compared to full-term counterparts: a systematic review and meta-analysis
AIM: To evaluate the kidney health outcomes in ex-preterm children and adolescents (≤32 weeks of gestation).
DESIGN: A systematic literature search through the major databases (MEDLINE/PubMed, Scopus, and Web of Science). Observational studies comparing kidney markers in children ≤32 weeks of gestation with full-term counterparts were included.
difference in Cr-eGFR
difference in serum Cystatin C levels
OUTCOMES: Thirteen studies (16 reports) comprising 2112 participants were included. The very preterm group had higher serum cystatin C, lower Cr-eGFR, and higher SBP compared to the full-term peers.
Dokousli et al. 2025
CONCLUSION: This is the first systematic review and meta-analysis addressing the effect of prematurity ≤32 weeks of gestation on kidney function exclusively in children and adolescents. Elevated serum Cystatin C and SBP and lower Cr-eGFR may represent early markers of kidney dysfunction during childhood and adolescence.
