# Supplementary figures and images for: Kidney health outcomes in children born very prematurely compared to full-term counterparts: a systematic review and meta-analysis
Source: Pediatr Nephrol. 2025 May 26;41(1):61–72. doi: 10.1007/s00467-025-06797-z (PMC12686000; doi:10.1007/s00467-025-06797-z)

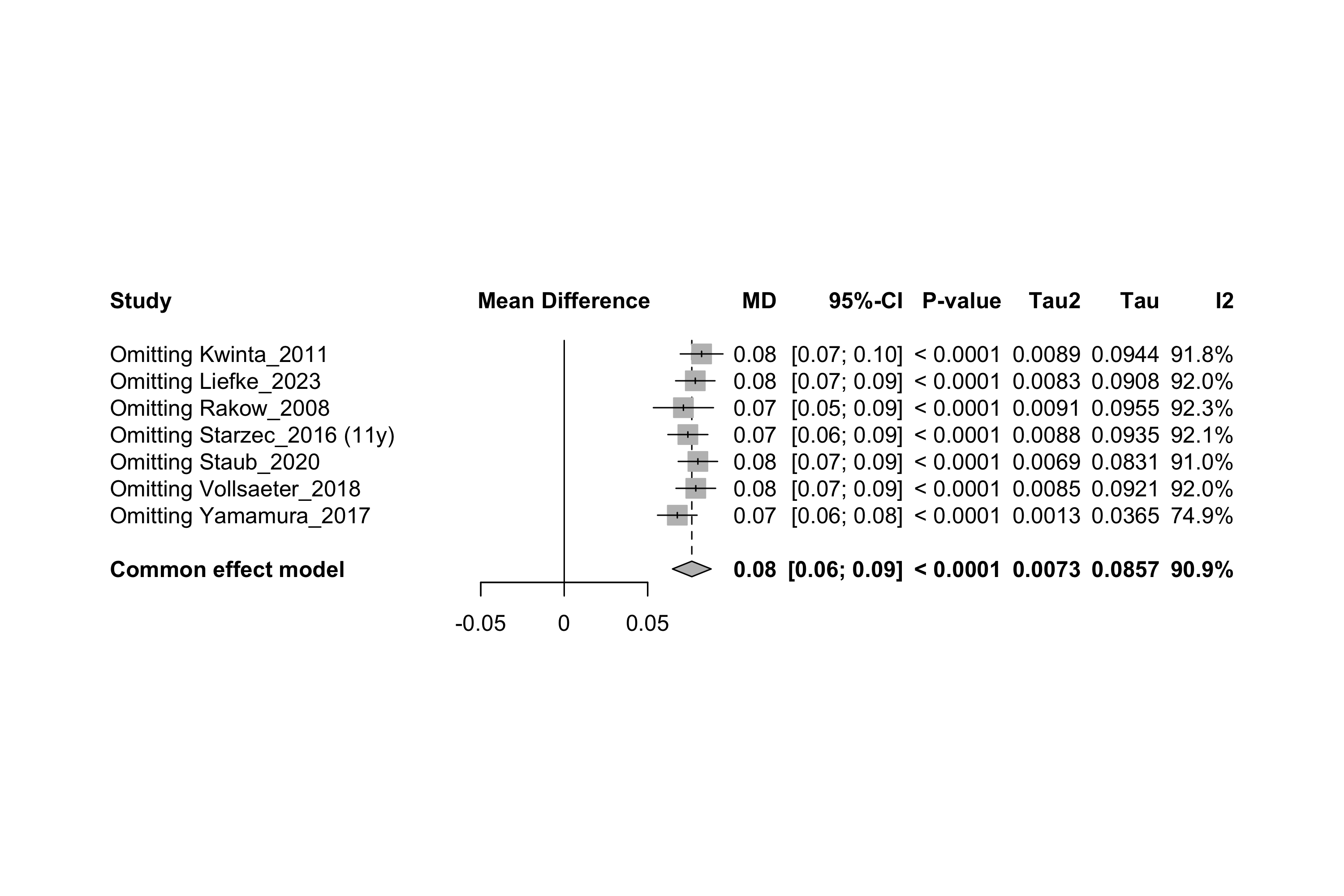

Supplement: Supplementary file 2 — Supplementary file2 Appendix Figure 1. Sensitivity analysis of the outcome of serum Cystatin C levels (PNG 296 KB) [file 467_2025_6797_MOESM2_ESM.png]

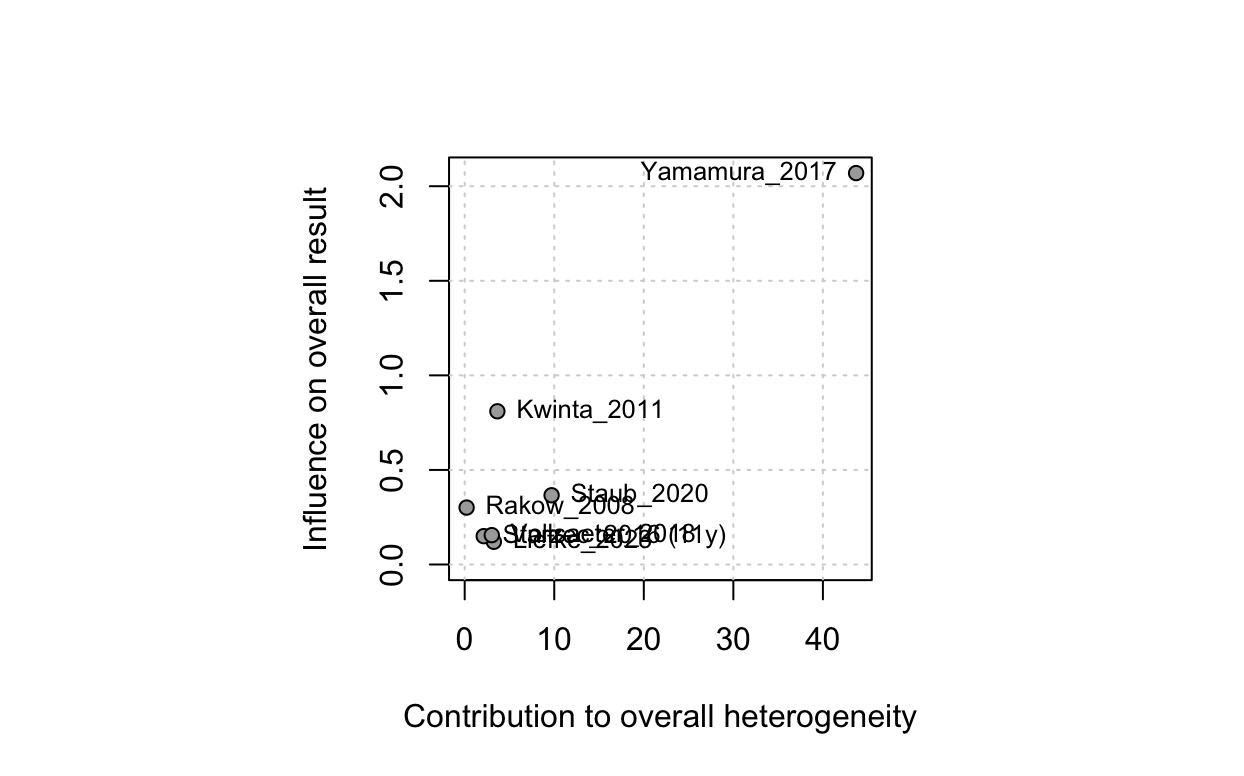

Supplement: Supplementary file 3 — Supplementary file3 Appendix Figure 2. Baujat plot for assessing the contribution of each study to overall heterogeneity in the outcome of serum Cystatin C levels (PNG 82 KB) [file 467_2025_6797_MOESM3_ESM.png]

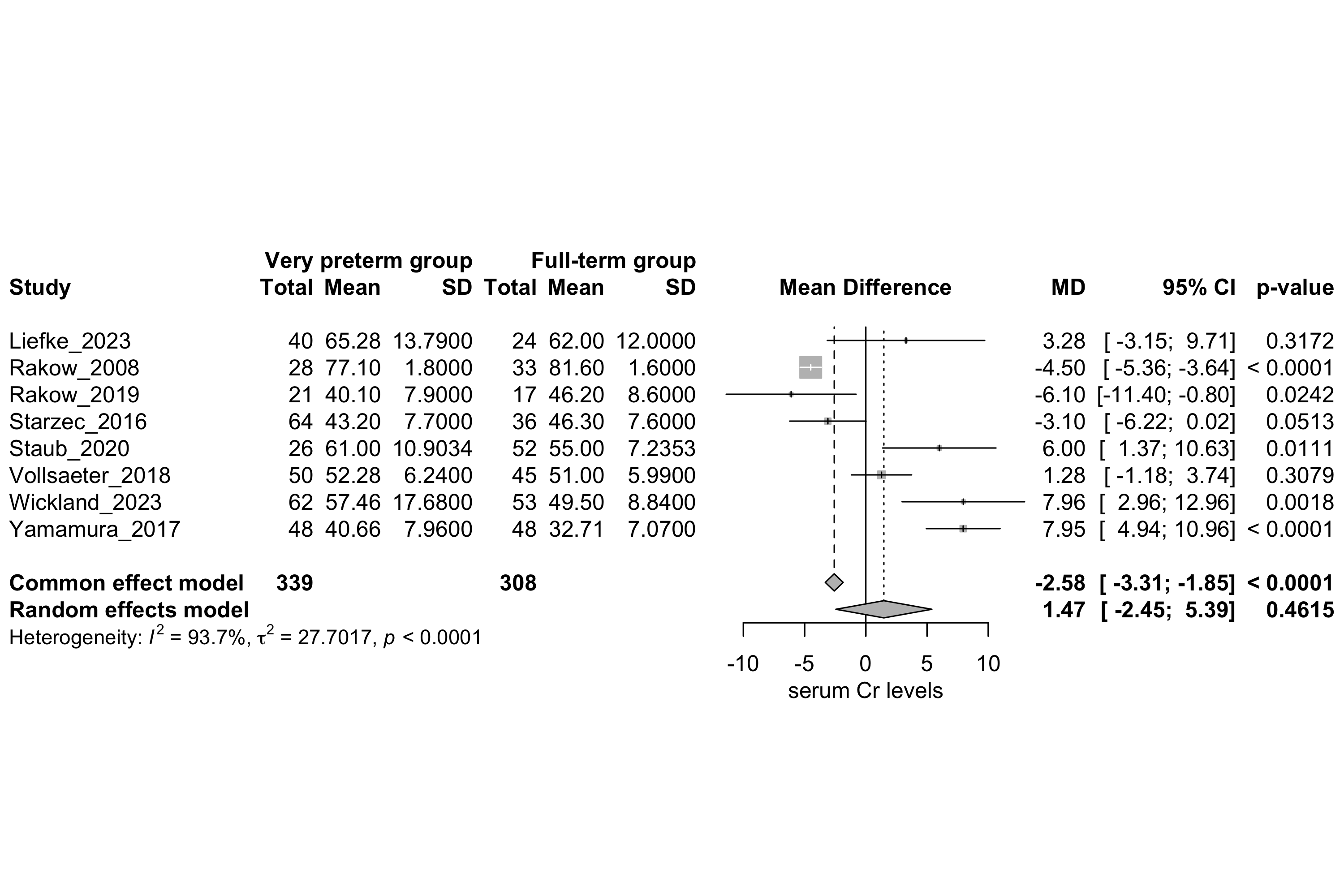

Supplement: Supplementary file 4 — Supplementary file4 Appendix Figure 3. Forest plot assessing the difference of serum creatinine levels between very preterm group vs. full-term group (PNG 344 KB) [file 467_2025_6797_MOESM4_ESM.png]

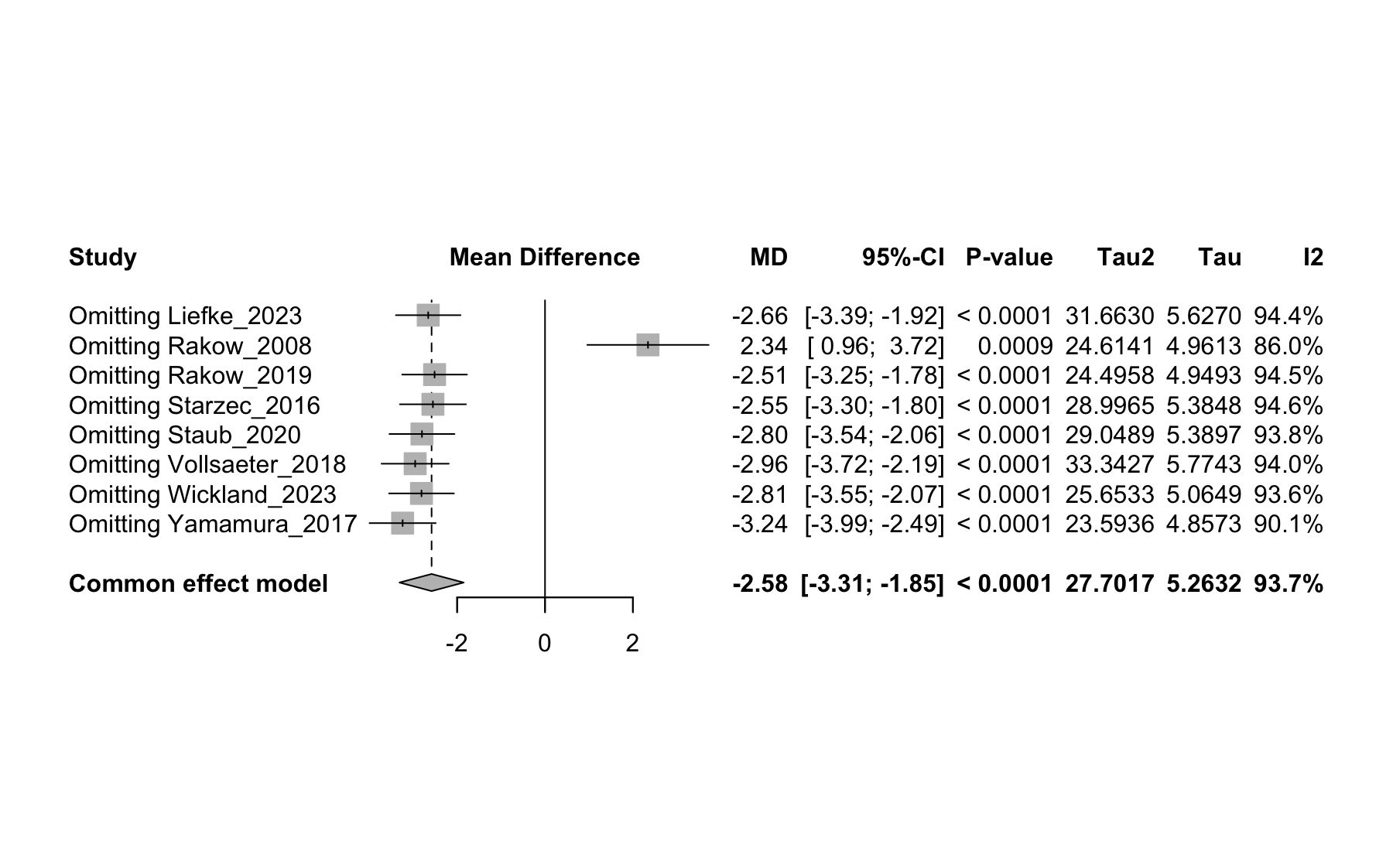

Supplement: Supplementary file 5 — Supplementary file5 Appendix Figure 4. Sensitivity analysis of the outcome of serum creatinine levels (PNG 189 KB) [file 467_2025_6797_MOESM5_ESM.png]

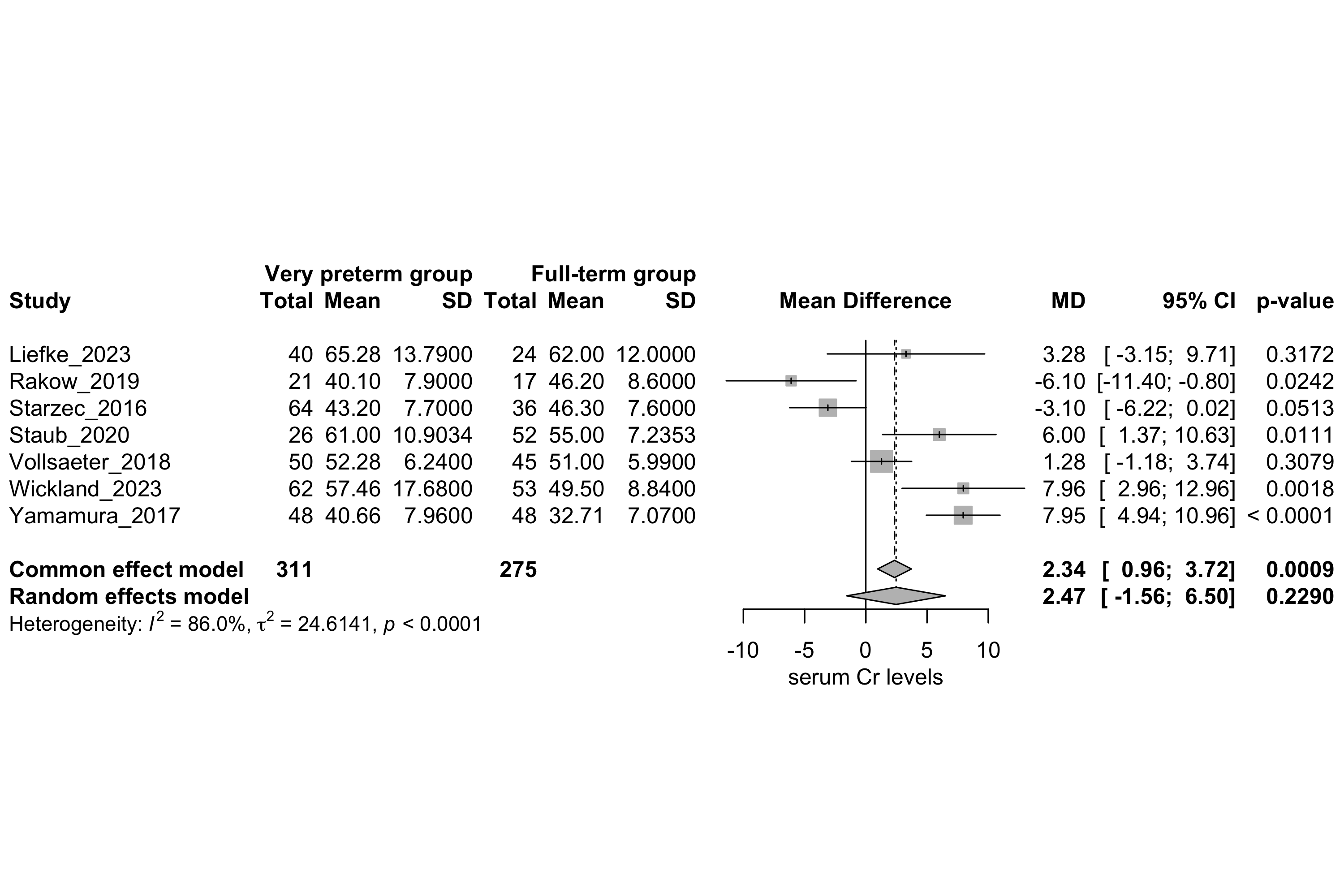

Supplement: Supplementary file 6 — Supplementary file6 Appendix Figure 5. Forest plot assessing the difference of serum creatinine levels between very preterm group vs. full-term group after sensitivity analysis (PNG 327 KB) [file 467_2025_6797_MOESM6_ESM.png]

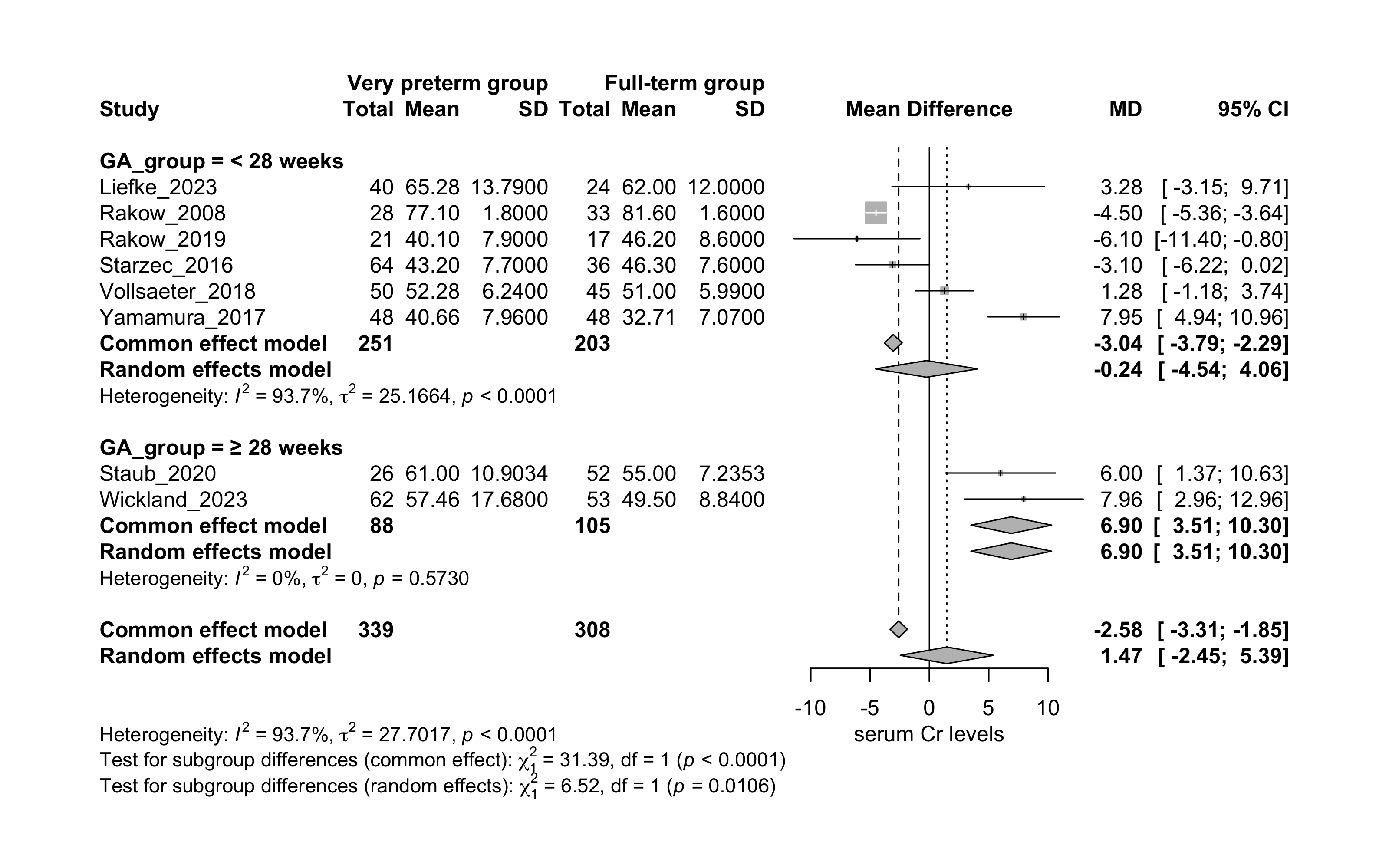

Supplement: Supplementary file 7 — Supplementary file7 Appendix Figure 6. Subgroup analysis of the outcome of serum creatinine levels by gestational age groups; GA: gestational age (PNG 510 KB) [file 467_2025_6797_MOESM7_ESM.png]

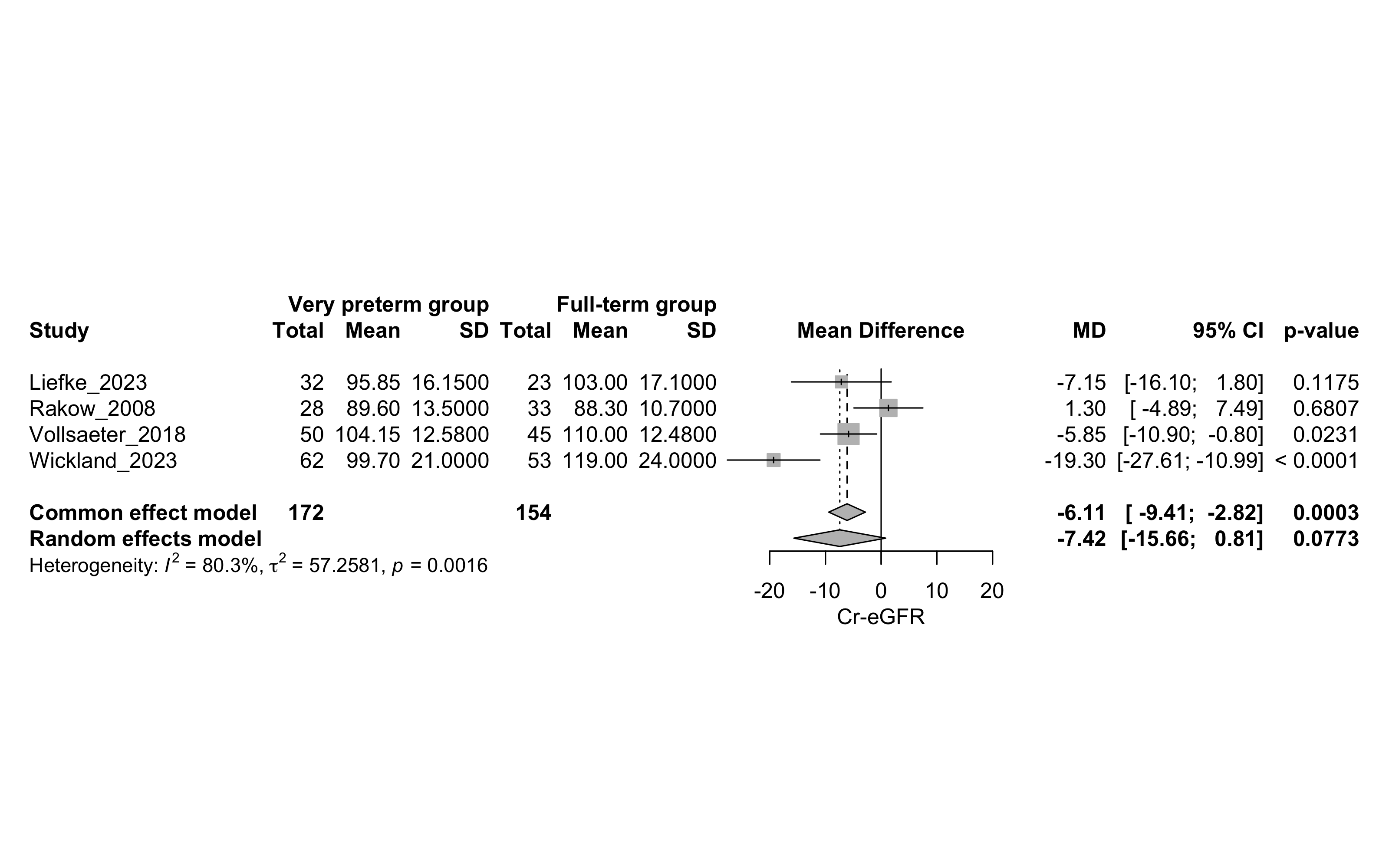

Supplement: Supplementary file 8 — Supplementary file8 Appendix Figure 7. Forest plot assessing the difference of Cr-eGFR between very preterm group vs. full-term group after sensitivity analysis; Cr-eGFR: Creatinine-estimated glomerular filtration rate (PNG 283 KB) [file 467_2025_6797_MOESM8_ESM.png]

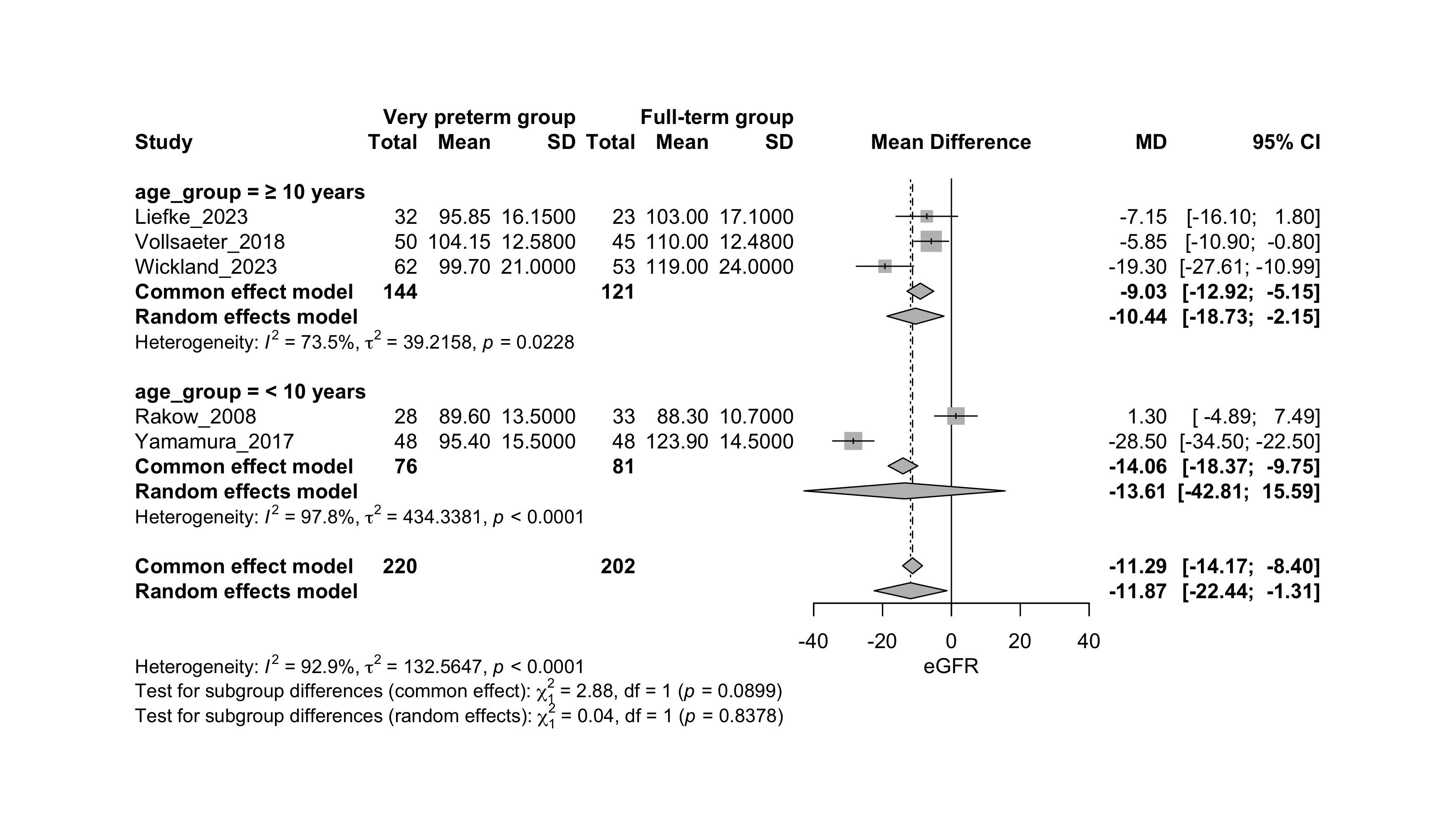

Supplement: Supplementary file 9 — Supplementary file9 Appendix Figure 8. Subgroup analysis of the outcome of Cr-eGFR by age at the time of assessment groups; Cr-eGFR: Creatinine-estimated glomerular filtration rate (PNG 470 KB) [file 467_2025_6797_MOESM9_ESM.png]

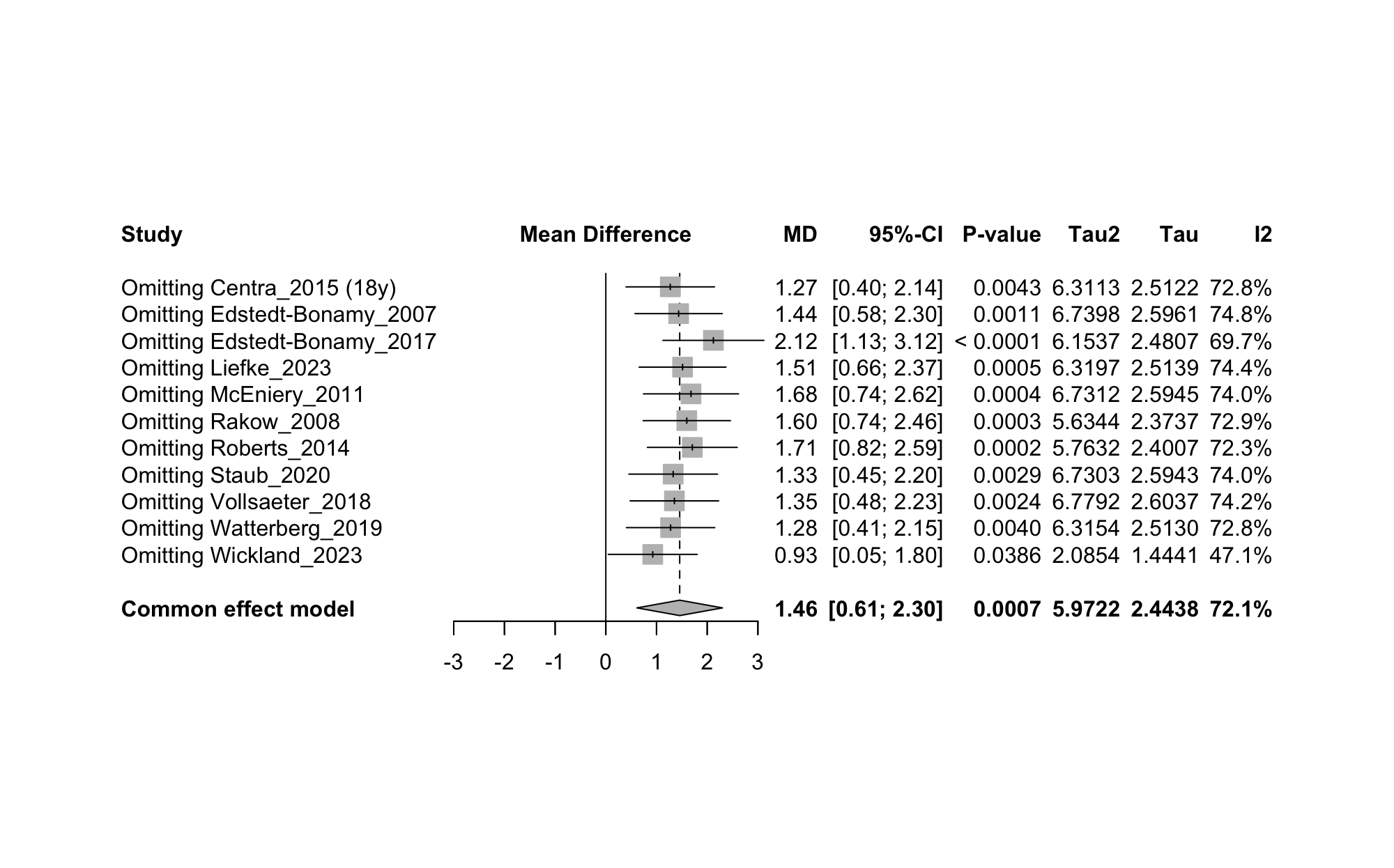

Supplement: Supplementary file 10 — Supplementary file10 Appendix Figure 9. Sensitivity analysis of the outcome of systolic blood pressure; SBP: systolic blood pressure (PNG 243 KB) [file 467_2025_6797_MOESM10_ESM.png]

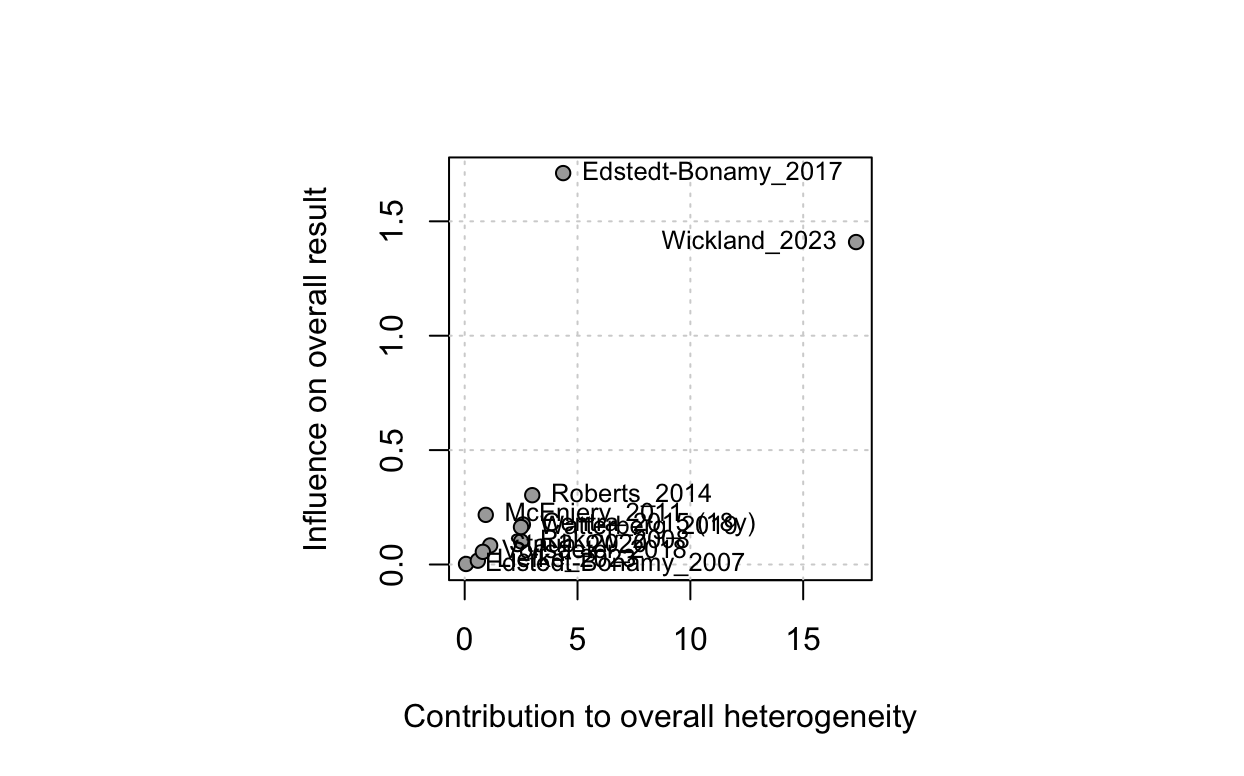

Supplement: Supplementary file 11 — Supplementary file11 Appendix Figure 10. Baujat plot for assessing the contribution of each study to overall heterogeneity in the outcome of systolic blood pressure; SBP: systolic blood pressure (PNG 91 KB) [file 467_2025_6797_MOESM11_ESM.png]

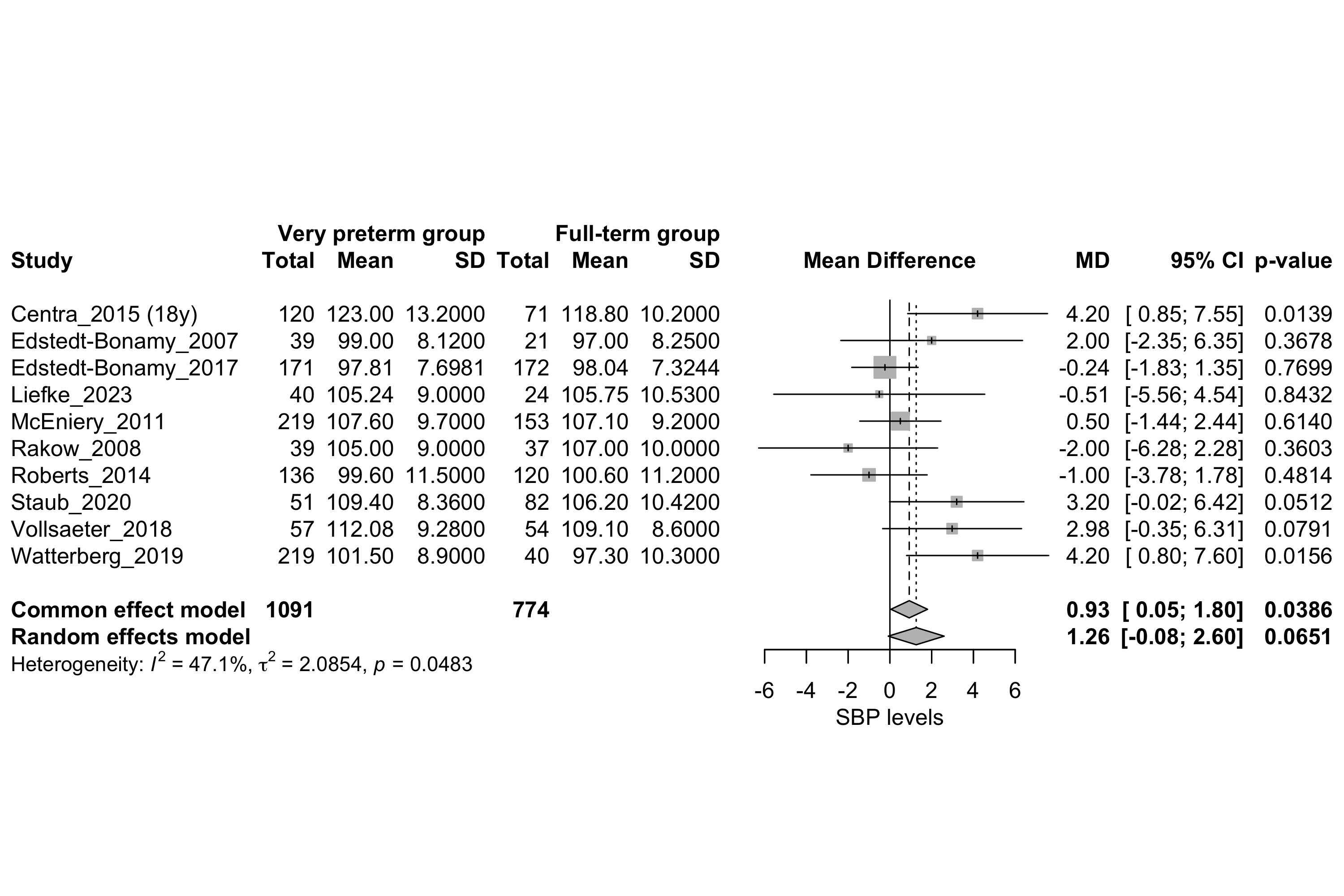

Supplement: Supplementary file 12 — Supplementary file12 Appendix Figure 11. Forest plot assessing the difference of systolic blood pressure levels between very preterm group vs. full-term group after sensitivity analysis; SBP: systolic blood pressure (PNG 393 KB) [file 467_2025_6797_MOESM12_ESM.png]

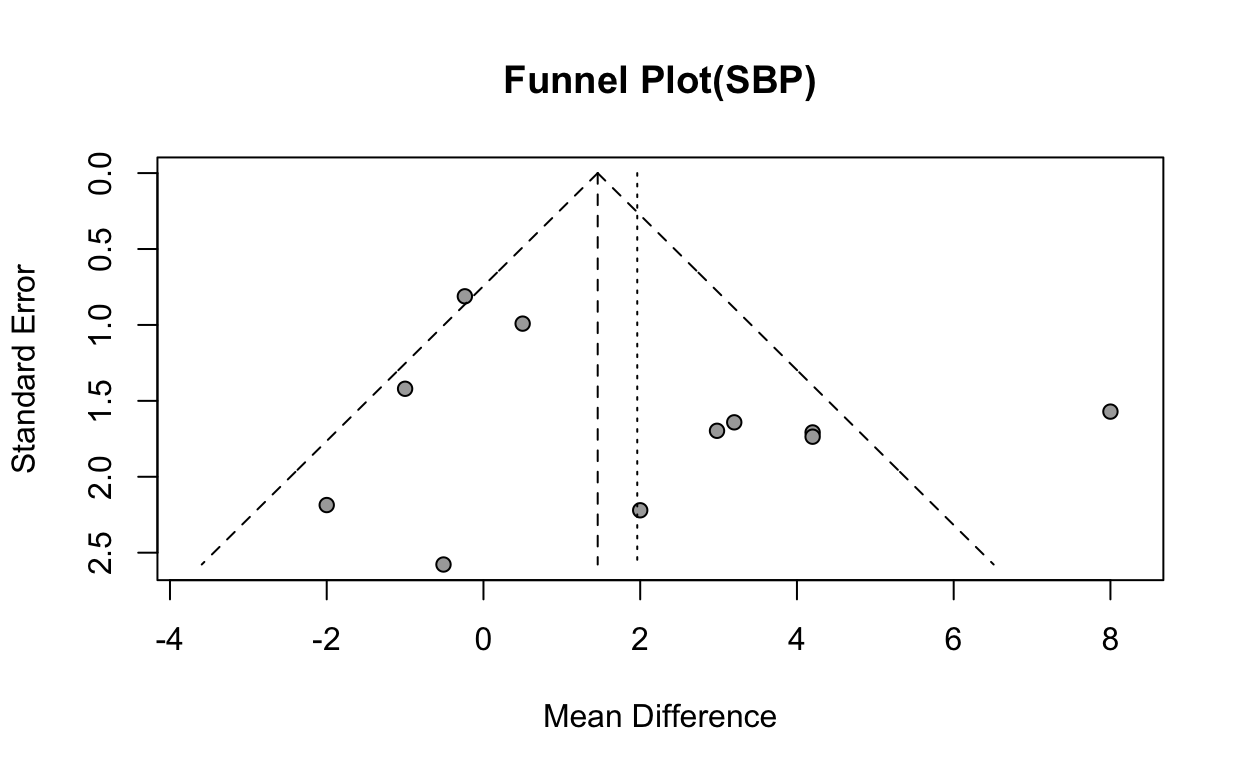

Supplement: Supplementary file 13 — Supplementary file13 Appendix Figure 12. Funnel plot for assessing publication bias (PNG 70 KB) [file 467_2025_6797_MOESM13_ESM.png]

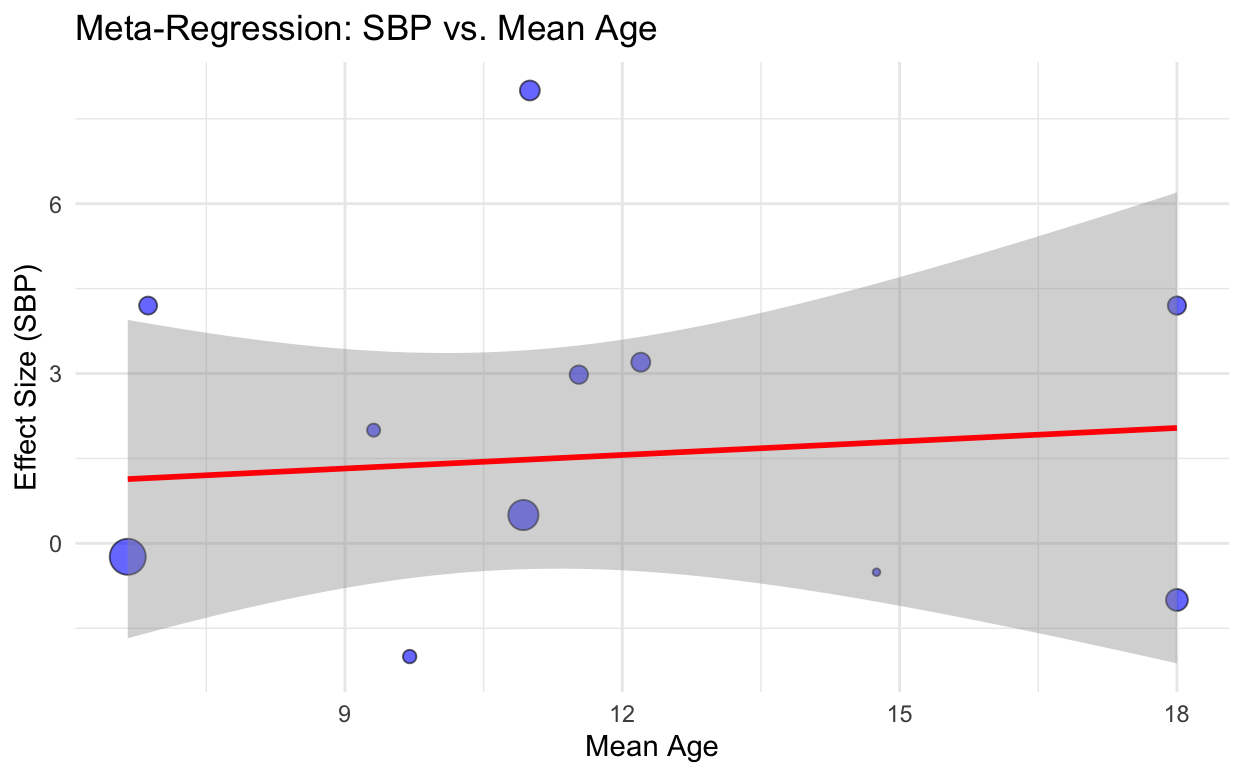

Supplement: Supplementary file 14 — Supplementary file14 Appendix Figure 13. Meta-regression analysis of systolic blood pressure effect size by mean age at assessment; SBP: systolic blood pressure (PNG 67 KB) [file 467_2025_6797_MOESM14_ESM.png]

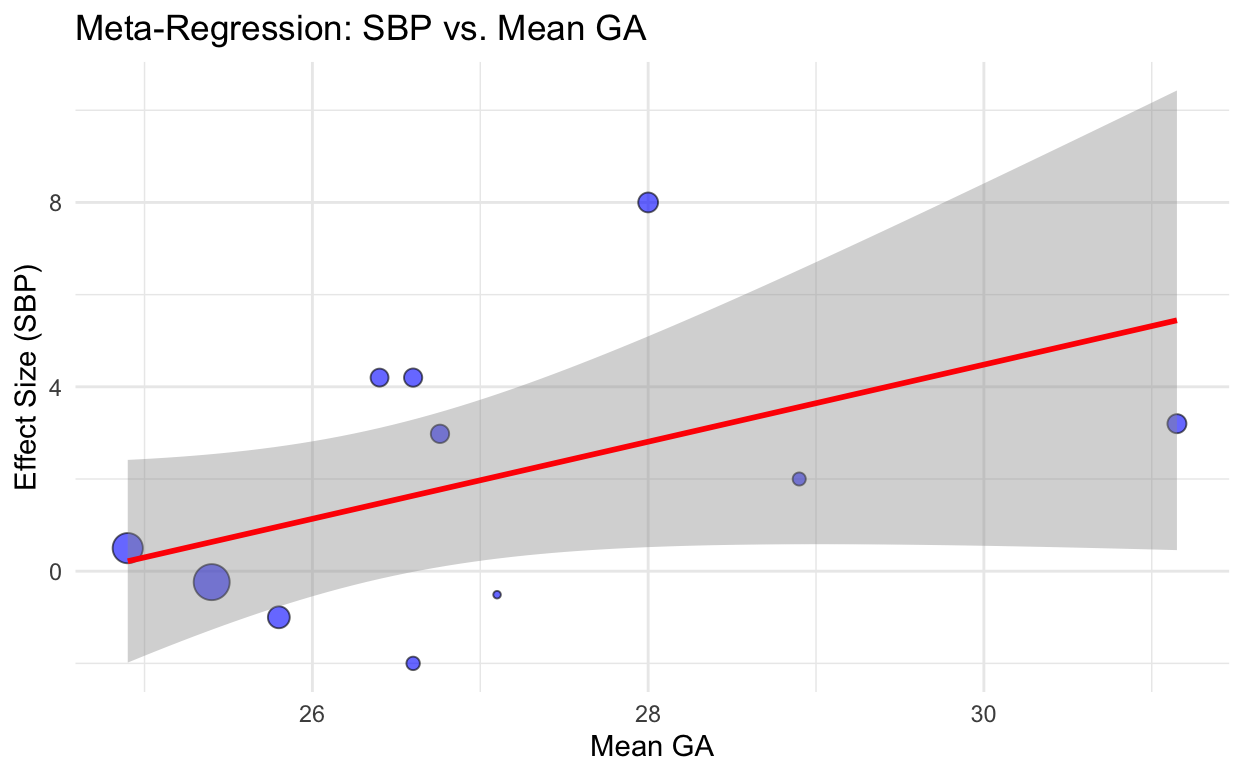

Supplement: Supplementary file 15 — Supplementary file15 Appendix Figure 14. Meta-regression analysis of systolic blood pressure by mean gestational age; SBP: systolic blood pressure; GA: gestational age (PNG 74 KB) [file 467_2025_6797_MOESM15_ESM.png]

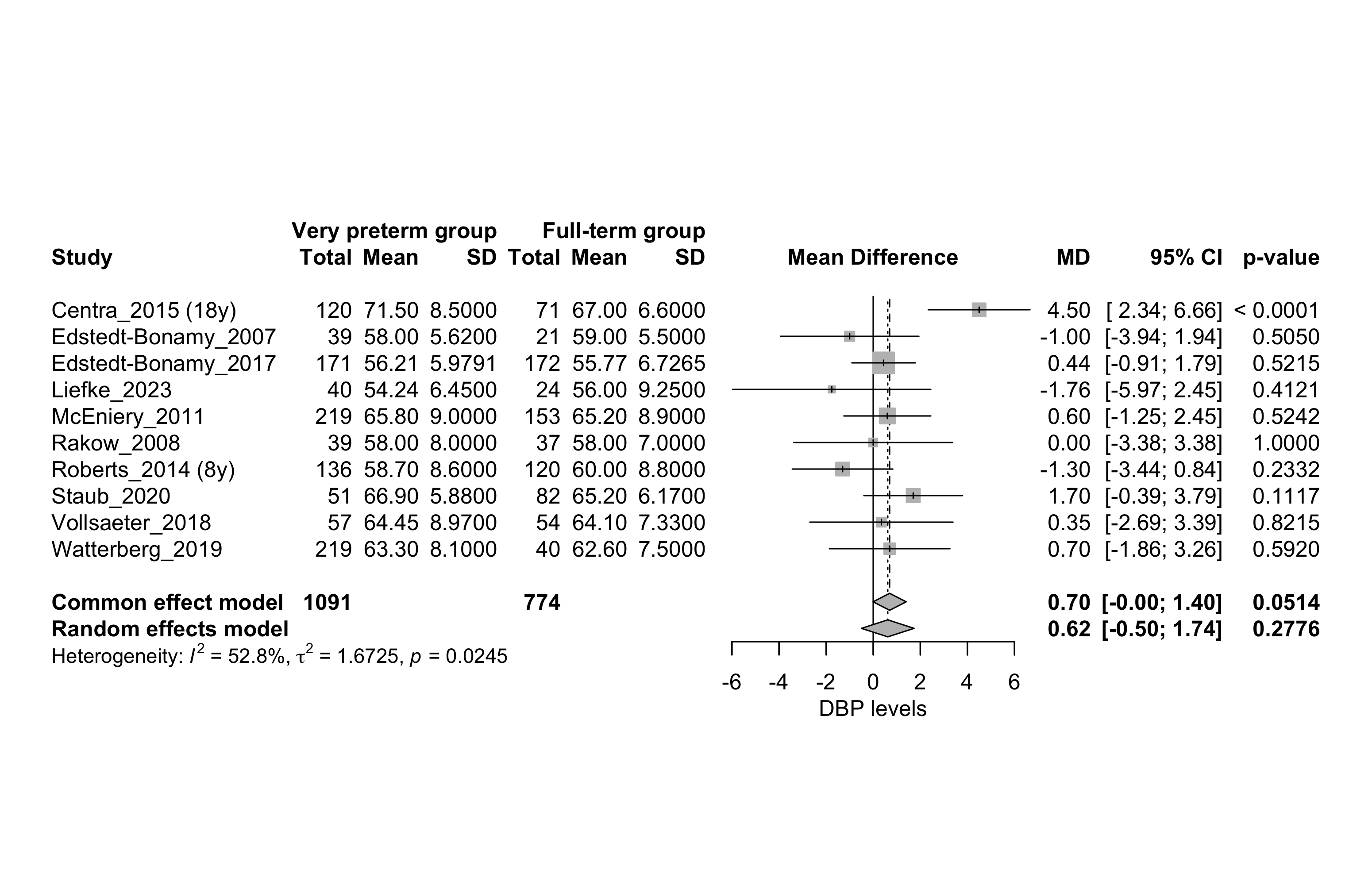

Supplement: Supplementary file 16 — Supplementary file16 Appendix Figure 15. Forest plot assessing the difference of diastolic blood pressure levels between very preterm group vs. full-term group; DBP: diastolic blood pressure (PNG 393 KB) [file 467_2025_6797_MOESM16_ESM.png]

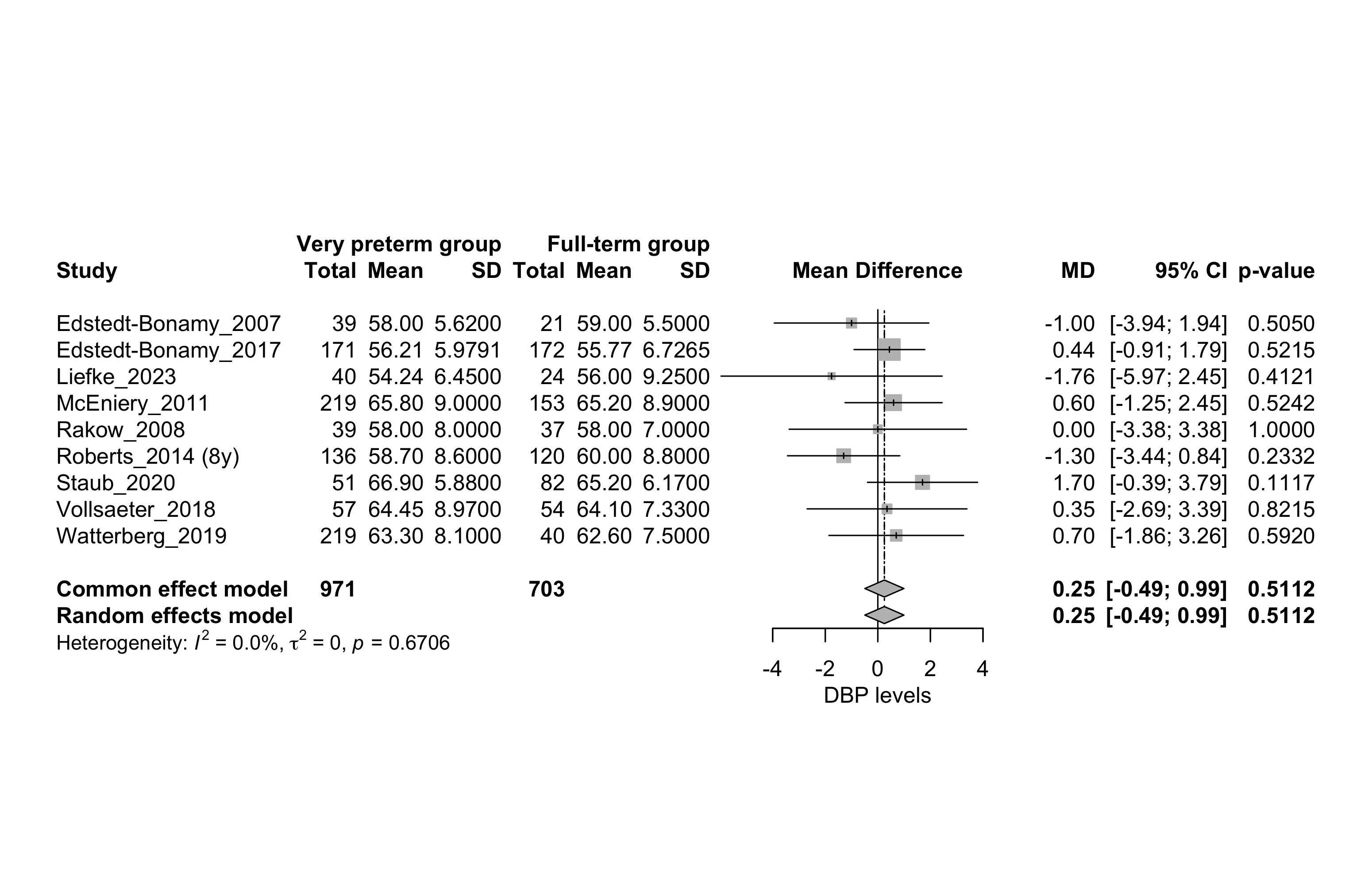

Supplement: Supplementary file 17 — Supplementary file17 Appendix Figure 16. Forest plot assessing the difference of diastolic blood pressure levels between very preterm group vs. full-term group after sensitivity analysis; DBP: diastolic blood pressure (PNG 369 KB) [file 467_2025_6797_MOESM17_ESM.png]
