# Supplementary material for: Kidney health outcomes in children born very prematurely compared to full-term counterparts: a systematic review and meta-analysis
Source: Pediatr Nephrol. 2025 May 26;41(1):61–72. doi: 10.1007/s00467-025-06797-z (PMC12686000; doi:10.1007/s00467-025-06797-z)
Supplement: Supplementary file 18 — Supplementary file18 (DOCX 33 KB) [file 467_2025_6797_MOESM18_ESM.docx]

Table 1. Excluded studies

| Author/year | Journal | Reason for exclusion |
| --- | --- | --- |
| Liefke 2023 | Pediatric Nephrology | Insufficient/inappropriate data |
| Hochmayr 2023 (EVA-Tyrol study) | BMC Cardiovascular Disorders | Wrong population |
| Vale 2024 | Braz.J. Nephrol. | Wrong population |
| Prinz 2023 | PLOS Medicine | Wrong population |
| Pearson 2022 | Precis Nutr (Precision Nutrition) | Wrong population |
| Skogastierna 2022 | Acta Paediatrica | Wrong population |
| Yang 2022 | Wei sheng yan jiu | No English language |
| Maguolo 2021 | Nutrition, Metabolism & Cardiovascular Diseases (j.numecd) | Wrong population |
| Kaze 2020 | BMC Nephrology | Wrong population |
| Sanderson 2020 | frontiers in Pediatrics | No/wrong control group |
| Ordonez-Diaz; Perez-Navero 2020 | frontiers in Pediatrics | Wrong population |
| Ordonez-Diaz; Gil-Campos 2020 | nutrients | Wrong population |
| Park 2020 | BMC Pediatrics | Wrong population |
| Umboh 2019 | International Journal of Nephrology | Wrong population |
| Weres 2019 | Int.J.Environ. Res. Public Health | Wrong population |
| Horie 2019 | Pediatrics International | No/wrong control group |
| Wang 2019 | Int.J.Environ. Res. Public Health | Insufficient/inappropriate data |
| Zamir 2019 | Pediatric Research | No/wrong control group |
| Vohr 2018 | PEDIATRICS | No/wrong control group |
| Stock 2018 | The Journal of Pediatrics | Wrong population |
| Tan 2018 | Journal of Human Hypertension/Springer Nature | Wrong population |
| Vohr, Heyne 2018 | The Journal of Pediatrics (J Pediatr.) | No/wrong control group |
| Kowalski 2018 | Journal of Hypertension | Wrong population |
| Gamboa Delgado 2017 | Nutricion hospitalaria | No English language |
| Ferreira 2018 | Jornal de Pediatria | Wrong population |
| Rhone 2017 | Pediatrics International | Wrong population |
| Ramirez-Velez 2017 | Early Human Development | Wrong population |
| Souza 2017 | PLOS ONE | Wrong population |
| Khalsa 2016 | Pediatr Nephrol | Wrong population |
| Toemen 2016 | Journal of Hypertension | Wrong population |
| Kowalski 2016 | The Journal of Pediatrics (J Pediatr.) | Wrong population |
| Gonzalez Stager 2016 | Revista Chilena De Pediatria | No English language |
| Alves 2016 | The Journal of Maternal-Fetal & Neonatal Medicine | Wrong population |
| Theodore 2015 | Hypertension | Wrong population |
| Juonala 2015 | Journal of Hypertension | Wrong population |
| Geremia 2015 | Italian Journal of Pediatrics | Wrong population |
| Skilton 2015 | The Journal of Pediatrics | Wrong population |
| Stroescu 2014 | Obesity research & clinical practice | Wrong population |
| Sipola-Leppänen 2014 | Pediatrics | Wrong population |
| Zhai 2014 | Zhonghua er ke za zhi | No English language |
| Zarrati 2013 | Iranian Red Crescent medical journal | Wrong population |
| Piemontese 2013 | La Pediatria medica e chirurgica : Medical and surgical pediatrics | No English language |
| Azadbakht 2014 | Nutrition | Wrong population |
| Chiolero 2014 | American journal of epidemiology (Am J Epidemiol.) | Wrong population |
| Nishizaki 2014 | Clinical and experimental nephrology (Clin Exp Nephrol) | Wrong population |
| Mullet 2014 | The Journal of pediatrics | Wrong population |
| Ponzio 2013 | European journal of pediatrics | Wrong population |
| Chaudhari 2012 | Indian pediatrics | Wrong population |
| Gopinath 2013 | Journal of human hypertension | Wrong population |
| Jouret 2011 | Hormone research in paediatrics | Wrong population |
| Fattal-Valevski 2011 | The Israel Medical Association journal : IMAJ | Wrong population |
| Belfort 2012 | Journal of perinatology | No/wrong control group |
| Rossi 2011 | Journal of adolescent health | Wrong population |
| Belfort 2010 | Pediatrics | No/wrong control group |
| Odberg 2010 | Archives of disease in childhood. Fetal and neonatal edition | Wrong population |
| Szalapska 2010 | Pediatric endocrinology, diabetes, and metabolism | Wrong population |
| Bacchetta 2009 | Archives de pediatrie | No English language |
| Lurbe 2009 | Hypertension | Wrong population |
| Salgado 2009 | Arquivos brasileiros de cardiologia | Wrong population |
| Salgado; Jardim 2009 | Clinical nephrology | Wrong population |
| Puddu 2009 | The journal of maternal-fetal & neonatal medicine | Wrong population |
| Hirschler 2008 | Archives of medical research | Wrong population |
| Strufaldi 2009 | European journal of pediatrics | Wrong population |
| Hua 2007 | Zhonghua nei ke za zhi | No English language |
| Covelli 2007 | The Journal of cardiovascular nursing | Wrong population |
| Bayrakci 2007 | The Journal of pediatrics | Wrong population |
| Bracewell 2008 (EPICure study) | Archives of disease in childhood. Fetal and neonatal edition | Wrong population |
| Franco 2007 | Hypertension | Wrong population |
| Rossi 2006 | Archives des maladies du coeur et des vaisseaux | No English language |
| McCormick Covelli 2006 | Issues in comprehensive pediatric nursing | Wrong population |
| Kniazewska 2006 | Przeglad lekarski | No English language |
| Elgen 2005 | Acta paediatrica | Wrong population |
| Pileggi 2005 | Journal of paediatrics and child health | Wrong population |
| Rodríguez-Soriano 2005 | Pediatric nephrology | Wrong population |
| Primatesta 2005 | Hypertension | Wrong population |
| Cheung 2004 | Archives of disease in childhood | Wrong population |
| Vaisbich 1999 | Jornal de pediatria | No English language |
| Pulzer 2001 | Metabolism: clinical and experimental | Wrong population |
| Spencer 2001 | American journal of kidney diseases | Wrong population |
| Harmoinen 2000 | Pediatric nephrology | No/wrong control group |
| Martin 2000 | Circulation | Wrong population |
| Finney 2000 | Archives of disease in childhood | No/wrong control group |
| Erhardt 1999 | Orvosi hetilap | No English language |
| Yiu 1999 | American journal of kidney diseases | Wrong population |
| Pharoah 1998 | Archives of disease in childhood. Fetal and neonatal edition | Wrong population |
| Uiterwaal 1997 | Hypertension | No/wrong control group |
| Donker 1997 | American journal of epidemiology | No/wrong control group |
| Hack 1993 | The Journal of pediatrics | Wrong population |
| Vanpée 1992 | The Journal of pediatrics | Wrong population |
| Karayel 2023 | Trends in Pediatrics | Wrong population |
| Prieto-Peña 2021 | Revista Medica Electronica | No English language |
| Rodríguez 2021 | Revista de Nefrologia, Dialisis y Trasplante | No English language |
| Hirschler 2020 | Revista de la Sociedad Argentina de Diabetes | No English language |
| Souza 2019 | Journal of Pediatrics | No/wrong control group |
| Kaźmierczak-Pilch 2019 | Pediatria i Medycyna Rodzinna | Wrong population |
| Abrarov 2019 | Pediatriya - Zhurnal im G.N. Speranskogo | No English language |
| Rafikova 2019 | Neonatology | No English language |
| Haas 2015 | International Journal of Preventive Medicine | Wrong population |
| Ranke 2016 | Journal of Pediatric Endocrinology and Metabolism | No/wrong control group |
| Arrocha 2015 | Revista Habanera de Ciencias Medicas | No English language |
| Derraik 2015 | PLoS ONE | Wrong population |
| Álvarez Mingorance 2011 | Acta Pediatrica Espanola | No English language |
| Relton 2008 | Journal of Human Hypertension | Wrong population |
| Nair 2009 | Indian pediatrics | Wrong population |
| Pérez Caballero 2004 | Revista Cubana de Medicina | No English language |
| Barros 1999 | International Journal of Epidemiology | Wrong population |
| Uckardes 2018 | Pediatric Nephrology | No full text/meeting abstract |
| Hack 1990 | Pediatric Research | No full text/meeting abstract |
| Staub 2016 | European journal of pediatrics | No full text/meeting abstract |
| Bacchetta 2008 | Pediatric Nephrology | No full text/meeting abstract |
| Özden 2010 | Pediatric Nephrology | No full text/meeting abstract |
| al Salmi 2007 | Pediatric Nephrology | No full text/meeting abstract |
| Brathwaite 2023 | Pediatric Nephrology | Wrong population |
| Restrepo 2023 | Frontiers in pediatrics | Wrong population |
| Course 2023 | The Journal of Pediatrics | Wrong population |
| Chainoglou 2022 | Journal of Hypertension | Wrong population |
| Landmann 2021 | Frontiers in pediatrics | Wrong population |
| Koutroumpa 2021 | Hormone research in paediatrics | Wrong population |
| Restrepo 2022 | Jornal de pediatria | Wrong population |
| Yun 2021 | BMC pediatrics | Wrong population |
| Torres-Canchala 2021 | Pediatric nephrology | Wrong population |
| Markopoulou 2021 | Pediatric research | Wrong population |
| Wei 2020 | Journal of the American Heart Association | Wrong population |
| South 2020 | Journal of human hypertension | Wrong population |
| Zhao 2019 | Clinical kidney journal | Wrong population |
| Holzer 2019 | Nutrition | Wrong population |
| South, Nixon 2019 | Pediatric nephrology | Wrong population |
| Yiallourou 2017 | Sleep | Wrong population |
| Raaijmakers 2017 | PloS one | Wrong population |
| Raaijmakers, Zhang 2017 | Hypertension | Wrong population |
| Posod 2016 | PloS one | Wrong population |
| Washburn 2015 | The Journal of pediatrics | Wrong population |
| Iacobelli 2007 | American journal of perinatology | Wrong population |
| Bonamy 2005 | Pediatric research | Wrong population |
| Brouwer 2024 (PEPC study) | CHILDHOOD OBESITY | Overlapping population |
| South 2018 | JOURNAL OF HYPERTENSION | Wrong population |
| Kwinta 2013 | Neonatology | Overlapping population |
| Bergmann 2017 | Journal of perinatal medicine | No outcome of interest |
| Park 2019 | Journal of Korean medical science | Small sample size |
| Vohr 2010 | Acta paediatrica | Wrong population |
| Bacchetta 2009 | Kidney international | No/wrong control group |
| Mohlkert 2018 | Journal of the American Heart Association | Overlapping population |
| Lee 2014 | Archives of disease in childhood | Small sample size |
| Steen 2015 | Acta paediatrica | Insufficient/inappropriate data |
| Bolton 2012 | The Journal of pediatrics | Overlapping population |
| Chan 2010 | International journal of pediatrics | Wrong population |
| Hack 2014 | PEDIATRIC RESEARCH | No outcome of interest |
| Johansson 2007 | Journal of internal medicine | Overlapping population |
| Liefke, Steding-Ehreborg 2023 | Pediatric research | Overlapping population |
